# Cell Heterogeneity Uncovered by Single-Cell RNA Sequencing Offers Potential Therapeutic Targets for Ischemic Stroke

**DOI:** 10.14336/AD.2022.0212

**Published:** 2022-10-01

**Authors:** Min Qiu, Jia-bin Zong, Quan-wei He, Yu-xiao Liu, Yan Wan, Man Li, Yi-fan Zhou, Jie-hong Wu, Bo Hu

**Affiliations:** Department of Neurology, Union Hospital, Tongji Medical College, Huazhong University of Science and Technology, Wuhan 430022, China.

**Keywords:** single-cell RNA sequencing, ischemic stroke, cellular heterogeneity, differentially expressed genes

## Abstract

Ischemic stroke is a detrimental neurological disease characterized by an irreversible infarct core surrounded by an ischemic penumbra, a salvageable region of brain tissue. Unique roles of distinct brain cell subpopulations within the neurovascular unit and peripheral immune cells during ischemic stroke remain elusive due to the heterogeneity of cells in the brain. Single-cell RNA sequencing (scRNA-seq) allows for an unbiased determination of cellular heterogeneity at high-resolution and identification of cell markers, thereby unveiling the principal brain clusters within the cell-type-specific gene expression patterns as well as cell-specific subclusters and their functions in different pathways underlying ischemic stroke. In this review, we have summarized the changes in differentiation trajectories of distinct cell types and highlighted the specific pathways and genes in brain cells that are impacted by stroke. This review is expected to inspire new research and provide directions for investigating the potential pathological mechanisms and novel treatment strategies for ischemic stroke at the level of a single cell.

## 1. Introduction

Stroke, including ischemic stroke (IS) and hemorrhagic stroke (HS), is the primary cause of death among adults, worldwide, and is characterized by high disability, morbidity, and mortality [1]. Among them, 75-80% of the cases are of IS due to the interruption of cerebral blood flow. It imposes substantial economic and social burdens especially among the elderly [2, 3]. The neurovascular unit (NVU) is comprised of neurons, glial cells, and vascular-associated cells. The current understanding of pathological mechanisms after stroke is based on the multicellular interactions within the NVU and peripheral immune cells [4], both involved in the evolution of damage to the blood-brain barrier (BBB), glial reactions, neuronal cell death, and immune cell infiltration [5-7].

Nevertheless, NVU and peripheral immune cells exhibit highly heterogeneous responses to IS [8], therefore, posing a challenge for evaluating the accura

te roles of the specific cell subpopulations during IS [9]. Recently, rapidly evolving high-throughput sequencing technologies including single-cell RNA sequencing (scRNA-seq) have aided the construction of a comprehensive reference map of cell transcriptional states and facilitated further studies on cell heterogeneity in stroke conditions at the level of a single cell [10-15]. scRNA-seq may thus contribute to identifying new potential biomarkers, therapeutic targets, and elucidating the molecular underpinnings underlying the pathological processes in IS[16].

In this review, we have provided an overall introduction to scRNA-seq technology and its promising application in studies related to stroke. Moreover, we have summarized the multiple subpopulations within each cell type and the differentially expressed genes (DEGs) in each of the cellular subpopulations implicated in stroke as evidenced by scRNA-seq. We hope that this review will provide novel insights for further discoveries of novel specific biomarkers and signaling pathways involved in the pathological processes and effective treatment modalities for IS.

## 2. scRNA-seq of cell transcriptome in stroke

**Figure 1.**
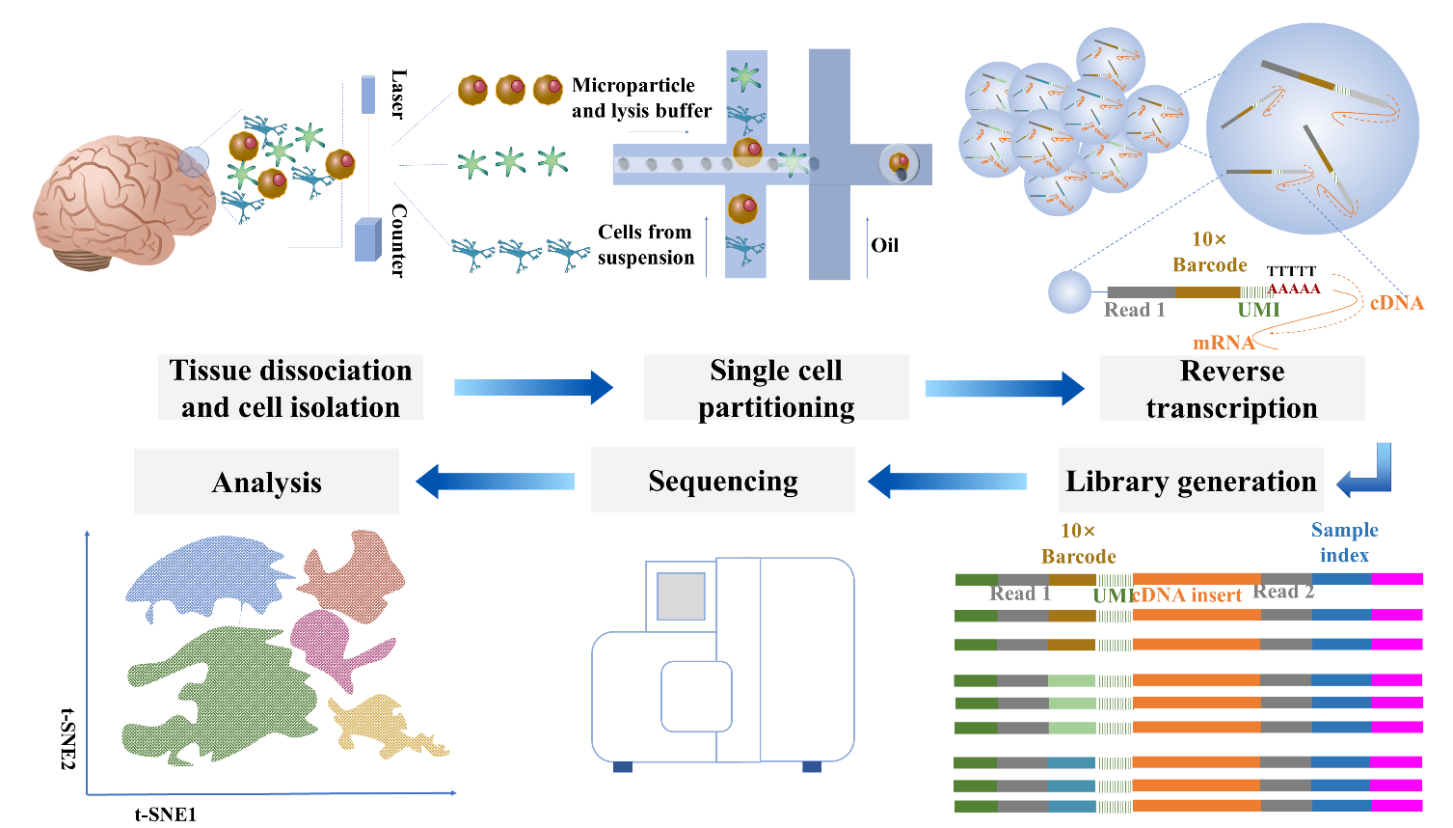
**Workflow of scRNA-seq**. Single-cell RNA sequencing begins with the dissociation of the tissue of interest for isolating single cells. The dissociated cells are loaded onto a cartridge or microfluidic chip for compartmentalization into nanoscale compartments. Each nanoscale compartment is attached to a unique oligonucleotide sequence following cell identification. Then the RNA from single cells is reverse transcribed and PCR amplified to obtain cDNA for library generation. Next, the pooled library is sequenced on the Illumina platform. Finally, the users can analyze the results of scRNA-seq.

High-throughput scRNA-seq has enabled an unbiased determination of cell heterogeneity at a high resolution and the identification of cellular markers by the assessment of the transcriptomic profiles at the single-cell level [17], thus combining neuroscience with computational biology [18, 19]. scRNA-seq unveils molecular taxonomy and gene regulatory mechanisms of brain cells in neurological disease conditions with unprecedented precision and depth [20-23]. The workflow of scRNA-seq includes tissue dissociation, cell isolation, single-cell partitioning, reverse transcription, library generation, sequencing, and finally the analyses [24]. The general workflow of scRNA-seq is illustrated in Figure 1.

Given that the current treatment strategies for stroke are limited to a narrow treatment window and have failed to address progressive neuronal degeneration and loss of function, scRNA-seq is performed to better understand the mechanisms underlying stroke and provide insights into novel potential therapeutic targets and biomarkers [25]. scRNA-seq provides new insights into the complexity of stroke at the molecular level based on the identification of the new cellular subpopulations and potential disease-specific mechanisms [26]. Additionally, scRNA-seq can be used to compare distinct subpopulations and cell states during stroke which is expected to provide an opportunity to understand stroke mechanisms in detail [26, 27]. Notably, some previous studies elucidate the changes in DEGs, cellular composition with pseudo-differentiation, or pseudo-time tools at cellular resolution through the manipulation of a given gene [11, 13]. The representative DEGs between middle cerebral artery occlusion (MCAO) and sham groups, along with the related enriched pathways and processes are presented in Table 1. scRNA-seq can be used to examine the regenerative potential of the brain [12]. Bobadilla et al. have confirmed the presence of dormant neural stem cells (NSCs) that express diverse lineage-specific combinations of genes in IS. Additionally, they have identified that interferon (IFN)-γ signaling affects the transition from the resting to the activated state in stem cells following ischemic injury, thereby providing possible signaling module targets for regeneration post-stroke [12]. However, more studies are needed to translate the results of academic research into clinical practice for investigating novel therapeutic interventions. Moreover, peripheral cells may provide an alternative route to evaluate the mechanisms of a stroke [28]. Understanding the transcriptional landscape in peripheral cells may uncover biomarkers or signatures of neurological diseases. Additionally, blood cells from patients can be easily obtained and are also suitable for the scRNA-seq analysis [29, 30]. Interestingly, whether a subpopulation of peripheral blood can serve as a ‘window’ into the ischemic tissue, remains to be addressed [30-32].

**Table 1:** Cellular heterogeneity by scRNA-seq in IS.

Cell types	Refs.	Subclusters	DEGs in MACO group	Representative pathways and process
**Microglia**	[13]	14	Upregulated: Rcan1, Ccl4, Gadd45b, Cd83, Id2 Downregulated: Cx3cr1, P2ry12, Gpr34, Rgs2, Marcks	IL-17 signaling pathway and toll-like receptor signaling pathway, transcriptional mis-regulation

	[14]	5	Upregulated: Ccl12, Ccl7, Cd72, Lilrb4a, Spp1 Hpgd, Selplg, Gpr34, Siglech, P2ry12	Positive regulation of microglia cell migration, lysosome, apoptosis, neutrophil chemotaxis
**Astrocytes**	[13]	7	Upregulated: Fkbp5, Jund, Cdkn1a, Fos, Cyr61 Hbb-bs, Gm3764, Sox2, Hes5, Phkg1	Toll-like receptor signaling pathway, estrogen signaling pathway, MAPK signaling pathway, positive regulation of gene expression and metabolic processes
	[14]	2	Upregulated: Vim, AY036118, Gfap, Cdkn1a, Ccl4 Appl2, Itm2a, Ntm, Gria2, Dbp	Signal transduction respiratory electron transport, cellular responses to stress
**Oligodendrocytes**	[13]	9	Upregulated: Htra1, Sgk3, Tma16, Phactr3, Cdkn1a Slc1a2, Phyhipl, Sytl2, Marcks, Fabp5	In response to L-glutamate and acidic amino acid transmembrane transporter activity, neurotransmitter transporter activity and oxygen-containing compounds
	[14]	2	Upregulated: Klk6, Phactr3, Gpd1, Tma16, Serpina3n Hs3st1, Omg, Lpar1, 1700047M11Rik, Hebp1	Regulation of neuron projection and apoptotic process, glial cell differentiation, cytokine-mediated signaling pathway
**Neurons**	[13]	6	Upregulated: Ay036118, Ccl4, Gfap, Mt1, Mt2 Cd24a, Basp1, Stmn2, Nr2f1, Gm17750	Nervous system development, cell differentiation, neurogenesis
**Endothelial cells**	[13]	5	Upregulated: Lcn2, Mt2, Mt1, Akap12, Tmem252 Tgfb2, Slc16a1, Car4, Tfrc, Hmcn1	Cyclic nucleotide metabolism, glutathione metabolism, ROS detoxification and oxidative phosphorylation
	[14]	6	Upregulated: Tmem252, Lcn2, Lrg1, Plat, Ctla2a Itm2a, Ifit3, Spock2, Cxcl12, Rps27rt	Transport of small molecules, anion transport, regulation of cell death, cellular response to chemical stimulus
**Pericytes**	[14]	3	Upregulated: Ccl11, Saa3, Timp1, Il11, Ednrb Tsc22d1, Pltp, Cxcl12, Itm2a, Dbp	Transport of small molecules, response to peptide, regulation of secretion
**Vascular muscle smooth cells**	[14]	6	Upregulated: Rasl11a, Sdc4, Cdkn1a, Ccl4, Ifitm1 Myh11, Crim1, Lbh, Pln, Fbxl22	Relaxation of muscle, calcium ion transmembrane transport, cytokine-mediated signaling pathway, cellular response to chemical stimulus
**Fibroblast-like cells**	[14]	3	Upregulated: Ccl4, Angptl4 Nme2, Ifi27l2a, Dbp	Structural constituent of ribosome, myoblast differentiation
**Neutrophil**	[14]	4	Upregulated: Hcar2, Marcksl1, Cxcl2, Ccrl2, Cxcl3 Ly6g, Ngp, Cd177, Camp, Ltf	IL-1 signaling pathway, neutrophil degranulation
**Lymphocyte**	[14]	6	Upregulated: Lck, Gzma, Ccl3, Dusp2, Ccl5 Xcl1, Ly6d, Cd74, Lyz2, H2-Aa	Metalloprotease DUBs, leukocyte apoptotic process

Taken together, the rapidly evolving scRNA-seq technology provides an unprecedented single-cell-resolution brain map and has led to the accumulation of big data which can be used to further examine aberrations in neurological disorders [33]. Although at present, the knowledge of the brain and neurological disorders including stroke is far from adequate, overwhelming progress is expected in the coming decade with the development of single-cell sequencing technologies.

## 3. Microglial heterogeneity in IS

Microglial cells, the resident immune cells of the central nervous system (CNS), show an uneven distribution in the main CNS structures, along with a distinct ontogeny [34, 35]. Microglia exhibit regional heterogeneity, with higher densities in the white matter and the substantia nigra [36-38]. Microglia have important physiological functions in the human body [34], including monitoring infections in the brain parenchyma, dysfunction, and damage in motile and ramified processes [39]. Additionally, they rapidly react to the pathological conditions through morphological and functional changes [40], thereby improving the ability of the microglia to adapt and yield a context-specific phenotype [41]. Microglia have two diverse functional phenotypes, namely the M1 pro-inflammatory and M2 pro-regenerative [42, 43]. However, this is an oversimplified classification that points to the M1-M2 dichotomy that represents only two extreme activation states and fails to explain the diversity of different microglial subpopulations in the diseased states of the brain [44].

### 3.1 Role of microglia in IS

As the major immune cells, microglia undergo morphological and functional changes induced by the interruption of cerebral blood flow and energy supply during the ischemia [45]. At the onset of reperfusion, microglia within the ischemic penumbra are activated and extend their cell protrusions to neighboring vessels, thereby regulating endothelial cell functions [46]. Simultaneously, the adaptable and modifiable features of microglia also contribute to cellular repair and remolding after stroke [47]. Nevertheless, excessive activation of microglia due to damage-associated molecular patterns after IS result in the production of numerous proinflammatory cytokines, which can damage the BBB and brain cells, as well as influence the neurogenesis [48]. These distinct functions can be attributed to the different subclusters or phenotypes of microglia, including the destructive M1 and neuroprotective M2 types [49]. The lack of high-throughput unbiased approaches to evaluate microglial heterogeneity limits the study of the spatiotemporal distribution of their subpopulations in IS [50]. Nonetheless, the high-resolution scRNA-seq of microglia has provided new insights into the spatial and functional diversity of microglia, thereby expanding the knowledge of different phenotypic changes of microglia in ischemic stroke. In addition, scRNA-seq also contributes to the discovery of potential markers for the identification of different subclusters of microglia in IS [10].

### 3.2 Microglial heterogeneity in IS by scRNA-seq

In 2020, for the first time, Guo et al. had used scRNA-seq to investigate the cellular heterogeneity and molecular changes in mouse cortex penumbra at the acute stage of IS [13]. They used a model of MCAO and established a sham-operated group [13]. They have analyzed cellular heterogeneity during IS at a single-cell resolution between the MCAO and sham groups [13]. Microglial cells were found highly enriched in the MCAO group as compared to sham group [13], indicating that the microglia had undergone some significant alterations during IS. Another study reports a total of 275 DEGs in the microglia, the first among all the tested cells, between MCAO and sham group [14]. Together, these findings suggest the essential role of microglia in IS.

To better understand the microglial heterogeneity in IS, five unique microglial cell clusters were identified [14]. Microglia0 (MG0) was composed of cells mainly from the sham group, MG1 showed approximately equal percentages in both MCAO and sham groups, while MG2, 3, and 4 clusters were primarily composed of cells from the MCAO group. The highly expressed genes and possible functions of each microglial subcluster are presented in Table 2. MG1 cluster was verified by immunostaining and flow cytometry as CCL2^+^LGALS3^-^CXCL10^-^ and CCL2^hi^LGALS3^lo^CXCL10^lo^ [51, 52]. Notably, the MCP family of genes expressed in MG1 reportedly enhances chemotactic migration of peripheral immune cells into the infarcted areas of the brain [53]. The study indicates that the MG2 cluster expresses high levels of matrix metalloproteinases 12 (MMP12), which can damage the BBB after IS [54]. Additionally, ADAM8 is uniquely highly expressed in MG2 and is known to be involved in microglia-mediated neuroprotective effects through modulation of the TNF-R1 shedding [55].

In addition, five distinct transcription factor (TF) subsets were also identified using the single-cell regulatory network inference and clustering (SCENIC) method [56] and a regulatory network in microglia was obtained. In the MCAO group, MG0 and MG1 cells highly expressed cebpb, Egr1, and Fos in 3h after ischemic-reperfusion. These TFs are related to the immediate-early gene family [57]. Some candidate regulons, including Creb3, Ets2, and Sp3 have been identified in MG2. Ets2 reportedly induces the pro-inflammatory phenotype of endothelial cells and improves the expression of VCAM1, MCP1, and IL6 [58]. Sp3 can regulate NCX1 expression in IS [59]. TFs associated with type I IFN responses like Irf7, Stat1, and Stat2 are enriched in MG3. MG4 highly expresses Nfyb, Mybl2, and Ezh2. These indicate that MG4 is involved in cell mitosis, differentiation, and development [60]. Additionally, Dbp, Maf, and Klf2 are candidate TFs in MG0-3 identified in the sham group. However, Spi1 and Hif1a have been identified in MG0-4 from the MCAO group.

**Table 2:** Microglial heterogeneity by scRNA-seq in IS.

Subpopulations	Highly expressed genes in IS	Possible functions	Refs.
**MG0**	Core microglial markers: Siglech, Selplg, TMEM119, P2ry12, Olfml3, Gpr34	Mainly composed cells from the sham group	[14]

**MG1**	IER3, CCL7, CCL2, CCL12	Involving in pro-inflammatory reactions	[14]
**MG2**	MMP12, ADAM8, Fth1, Spp1, Lpl, Lilrb4, Lgals3	The most inflammatory subcluster in ischemic stroke Involving in shedding of TNF-R1 in microglia-mediated neuroprotective effects	[14]
**MG3**	Cxcl10, Irf7, Ifit3, Isg15	Involving in response to virus and interferon-beta	[14]
**MG4**	Stmn1, Top2a, Ube2c, Birc5	a proliferating subcluster of microglia	[14]

Another previous study divided 5108 microglia cells into 14 subpopulations from MCAO and sham groups (Table 1) [13]. Among the DEGs, Gadd45b could activate the TGF-β-smad3 pathway and reduce the infarct volume after IS [61, 62]. Gene set variation analysis (GSVA) was employed to investigate the responses of different microglial subclusters under hypoxic conditions [13]. Some genes that are highly expressed in the MCAO group have been proven subcluster-specific, such as Zbtb16 in subcluster 3; B4galt1, Plek, and Gadd45b in subcluster 4; Iba1 and P2y12 in subclusters 6 and 8; Csrnp1, Gadd45b, and Fkbp5 in subcluster 9, and Eif4ebp1 in subcluster 10 [13]. Inflammation-related pathways consisting of TNFα, IL-2, and IL- 6 and the hypoxia pathway are most enriched in subclusters 3, 4, 9, and 10 [13], indicating that these subclusters underwent an extreme inflammatory response during IS. In contrast, the Kras signaling pathway is enriched in subclusters 6 and 8, and thus, the two subclusters are more likely to survive after IS. Additionally, Arg1 and Ym1, the specific marker genes of M2 microglia [63, 64], are not completely expressed in the early period after a stroke [65], suggesting delayed differentiation of the M2 microglia. In addition, gene enrichment analysis suggests that the DEGs are extremely enriched in the IL-17 signaling pathway (NF-κB, Cebpb), toll-like receptor signaling pathway (Ccl4), and transcriptional misregulation (Cebpb, Id2, Gadd45b)[13].

Thus, the scRNA-seq of microglia indicates that these are prevalent in the early phases of IS and exhibit polarization. Several studies have attempted to regulate microglial activation in IS by inhibiting the M1 phenotype microglial activation, whilst stimulating microglial differentiation into the anti-inflammatory M2 phenotype to enhance restorative processes, axonal remodeling, neurogenesis, and angiogenesis [66, 67]. However, the traditional M1/M2 dichotomy oversimplifies the diversity of microglia due to substantial overlaps between the traditional M1/M2 subset. M1/M2 marker genes are not fully expressed in any of the above-mentioned microglial subclusters [13, 14]. Further application of scRNA-seq to investigate the mechanisms underlying microglial heterogeneity and multi-polarization during IS at single-cell resolution is thus imperative.

## 4. Heterogeneity in astrocytes in IS

Astrocytes, an abundant cell type in the CNS [68], are critical structural and functional components of the NVU [69]. Astrocytes are responsible for numerous housekeeping functions such as the BBB formation, regulation of cerebral blood flow, and cell communications [70-73]. In addition, they play critical roles in providing nutrition to adjacent neurons as most of the glycogen of the brain is stored in astrocytes [74]. Emerging evidence indicates two subpopulations of astrocytes, namely A1 and A2 [75]. A1 astrocytes, induced by proinflammatory factors like IL-1α and TNF-α, secrete components of the complement cascade. A1 astrocytes specifically express iNOS, regulated by the autocrine LCN-2 [76]. In contrast, protective A2 astrocytes can be induced by transcriptional activity of STAT3 in an effect to produce neurotrophic factors [68, 77].

### 4.1 Dual roles of astrocytes in IS

Astrocyte response to IS remains unclear. However, undoubtedly, they play important roles in immune responses after IS [78]. On the one hand, astrocyte inflammatory responses may exacerbate ischemic lesions during the acute phase after IS and the glial scar in the peri-infarct area may inhibit neuro-restoration during the late recovery stages [79, 80]. Inflammatory mediators like chemokines and cytokines are released by the BBB upon the destruction of the astrocyte gap junctions in IS [81]. On the other hand, astrocytes also contribute to neuroprotection by releasing neurotrophins and promoting angiogenesis, axonal remodeling, neurogenesis, along with the synaptogenesis [82-84]. Therefore, astrocytes are designated potential therapeutic targets for improving clinical outcomes following a stroke. Inhibition or further improvement in reactivity and function of astrocytes may depend on their location and specific subtypes, as also the time of ischemic lesion. Therefore, more research is required to deepen the understanding of the spatiotemporal dynamics of astrocyte transformation in IS. By employing scRNA-seq, the heterogeneity, distinct gene expression properties, and different transcriptomic patterns of astrocytes in IS can be studied, which could offer novel insights into stroke pathology and unveil potential drug targets [13].

### 4.2 scRNA-seq of astrocytes in IS

A total of seven subclusters among the 1,083 astrocytes can be detected in cases of IS using scRNA-seq (Table 1)[13]. Among them, subcluster 3 exhibits the greatest diversity in GSVA and pseudo-time trajectories [13]. The kras signaling pathway is enriched in subcluster 3, thereby contributing to neuroprotection and neuro-restoration during the post-acute phase of IS [13]. Additionally, thiamine metabolism and O-glycan biosynthesis functions in subcluster 3 are significantly different from those in other subclusters. Moreover, some specific genes are expressed in subcluster 3, including Cyr61, Sbno2, Socs3, and Klf4 [13]. Previous studies suggest that Socs3 may aggravate neuroinflammatory injury after stroke [85], while Klf4 confers vascular protection against cerebral ischemic injury [86]. SCENIC analysis suggests the overexpression of TFs, BTB and CNC homology 1 (Bach1), and CCHC-type zinc finger protein (Zcchc14), in the MCAO samples and upregulation of Ep300, Rbbp 5, and Mxi1 in the sham group [13].

**Table 3:** Gene markers and enriched processes in astrocyte (ASC) subclusters.

Subcluster	Gene markers	Enriched process	Refs.
**ASC1**	Rlbp1, C1ql2, Vcan, Pcdh15	Transcription regulation, synapse function/plasticity, cell proliferation/migration, cell adhesion	[11]
**ASC2**	Rbp1, Agt, Slc39a12, Gjp6, Sox9, Entpd2	Transcription regulation, synapse function/plasticity, neurotransmission, gap junction, cell differentiation, metabolism	[11]
**ASC3**	Rbp1, Txnip, Sox9	Transcription regulation, immune function, cell differentiation	[11]
**ASC4**	Nrsn2	Vesicle transportation	[11]
**ASC5**	Txnip, Kcnj8	Immune function, ion modulation/binding	[11]
**ASC6**	Enpp6, Vcan	Metabolism, cell proliferation/migration	[11]
**ASC7**	Txnip, Pln	Immune function, ion modulation/binding	[11]
**ASC8**	Gjp6, Txnip, Sox9, Entpd2, Enpp6, Plekhh1	Gap junction, immune function, cell differentiation, metabolism	[11]
**ASC9**	Vcan, Pcdh15	Cell proliferation/migration, cell adhesion	[11]
**ASC10**	Rbp1, Txnip, Sox9, Entpd2, Nme9	Transcription regulation, immune function, cell differentiation, metabolism, microtubule physiology	[11]

During the recovery phase of stroke, microglia and astrocytes play important roles in engulfing synapses through MEGF10 and MERTK associated pathways [87, 88], while exhibiting distinct phagocytic characteristics in IS and HS [88]. Recently, it has been reported that microglial-specific knockouts of MEGF10 or MERTK increase the dendritic spines and improve neurobehavioral outcomes in mice models of both IS and HS [11]. However, mice with astrocyte-specific knockout only show increased dendritic spines and improved outcomes in IS but not in HS conditions [11]. The differential analysis of transcriptional profiles using scRNA-seq demonstrates that 135 DEGs in astrocytes are downregulated in HS relative to IS [11]. A total of 10 subclusters from 1380 astrocytes with specific top gene markers representing distinct functional cell identities have been reported [11] (Table 3). The proportion of subcluster3 is about 20% in IS but <2% in HS, indicating that it may be responsible for the various phagocytic features of astrocytes in IS and HS. The analysis of 881 marker genes in subcluster3 demonstrates significant upregulation of processes related to the synapse pruning [11]. In microglia, analysis of scRNA-seq shows similar phagocytic patterns in IS and HS [11]. Collectively, this meaningful study indicates that scRNA-seq may contribute to the development of more precise therapies for the treatment of stroke in the future.

## 5. Heterogeneity in oligodendrocytes in IS

Oligodendrocytes (OLs) are found in both grey and white matter in the CNS [89]. OLs produce myelin, a multilamellar lipid structure that wraps around the neuronal axons, thereby accelerating axonal conduction velocity and transferring information across CNS [90]. OLs are derived from the oligodendrocyte progenitor cells (OPCs) [91]. The differentiation of OPCs into myelinating OLs can be promoted by neuronal activity [92], thus altering the internodal length and thickness of myelin. Previous studies also report that OPCs can proliferate and differentiate into myelinating OLs to facilitate the processes of recovery from the CNS injury [93]. Additionally, recent evidence indicates that OLs also possess some novel functions, including immune-related responses, supporting neuronal and axonal energy metabolism, regulating network activity by connecting the neuronal soma, interaction with the vasculature, and participating in processes of learning and memory [94, 95].

### 5.1 Role of OLs in IS

OLs are vulnerable to ischemic damage and do not have self-renewal capacity. They undergo necroptosis and apoptosis due to the release of toxic glutamate and ATP [96]. However, OLs also produce myelin sheaths on damaged axons during the late recovery phases of IS [97]. Oligodendrogenesis contributes to neuronal recovery and brain repair after stroke, contingent on the OPC proliferation, migration, and differentiation into mature OLs [98]. Previous studies also demonstrate that the number of OPCs increases in the penumbra but decreases in the core lesion after IS [99], suggesting the heterogeneity in OPCs. However, with altered potassium channel permeability, these become hypertrophic or die due to excitotoxicity [100]. Inflammatory cytokines are involved in oligodendrocytic modulation in cases of IS [96]. TNF-α and IFN-γ induce apoptosis in oligodendrocytes and inhibit OPC proliferation and differentiation [101, 102], while IL-4, IL-6, IL-11, and IL17 promote OL differentiation and survival, along with OPC differentiation during IS [103, 104]; additionally, other cells are also involved in neuroinflammation and interact with OLs or OPCs, including peripheral lymphocytes, microglia, and cerebral endothelial cells [105]. However, the heterogeneity of OLs in IS remains unclear and further investigations to evaluate the transcriptomic profiles of OLs at the single-cell level are needed.

### 5.2 scRNA-seq of OLs in IS

A recent study reports 9 subclusters among 1,154 OLs in IS by scRNA-seq analysis (Table 1)[13]. Overexpression of Anln, Arrdc2, and Klk6 are found in subcluster 4 [13]. Klf9, Plin4, Sgk1, and Sgk3 are upregulated in subcluster 9 [13]. This suggests that OLs may modulate neuronal and axonal energy metabolism in IS, however, this needs further validation. Moreover, GSVA demonstrates significant differences among the subcluster of OLs [13]. Subcluster 9 shows the highest enrichment of inflammatory-related pathways, such as the complement system, interferon-gamma responses, IL-2/stat5, IL-6/JAK/STAT3, and TNF-α/NF-κB signaling pathways [13]. Notably, the metabolic properties of each subcluster also exhibit differences, such as in enrichment of carbonic acid and creatine metabolism in subcluster 2 and steroid metabolism in subcluster 4 [13]. These indicate significant changes in metabolites of OLs during the acute phase of stroke. Although in-depth investigations on OLs in IS are limited, the recently reported scRNA-seq results suggest that a specific subcluster of OLs may play a critical role in neuronal and axonal energy metabolism, crucial for functional recovery.

## 6. Neuronal heterogeneity in IS

Cerebral ischemia deprives oxygen and glucose availability in the neuronal core thus inducing neuronal death [106]. In the surrounding penumbra, cells experience partial ischemic conditions and react to detrimental factors of oxidative stress, excitotoxicity, inflammation, and mitochondrial dysfunction; neurons in penumbra may undergo delayed apoptotic cell death, depending on the communication among different cells [107]. Neuronal death is a complicated process, as extensive communication networks are established among the neurons through synaptic transmission. Additionally, there are complex interactions among neurons, glial cells, and blood vessels in the NVU [4, 108, 109]. Failure in the synaptic processes and the NVU results in disconnection, trans-synaptic degeneration, and disruption of the BBB, causing neuronal dysfunction and eventually death [108]. With the expansion in the understanding of electrical synapses, tunneling nanotubes, and extracellular vesicles (EVs), neuronal communication networks have yielded greater complexity [110, 111]. Studies show that synaptic loss may result in neuronal death in the penumbra due to high energy demands [112]. EVs are reportedly involved in processes of neuroinflammation and neurodegeneration and can regulate the behavior of the recipient cells during IS [113].

As neuronal fates in cases of irreversible infarct core and salvageable ischemic penumbra are quite different, neurons in different areas may express a greater variety of receptor genes. Using scRNA-seq, investigating molecular determinants and lineage relationships of neurons after stroke are expected to yield novel therapeutic targets. A total of 246 neurons were subclustered from the MCAO or sham group into six subclusters in a recent study (Table 1)[13]. The GABAergic neurons showed the highest enrichment in subclusters 1 and 2, while dopamine (DA) neurons were mainly distributed in subcluster 5 [13]. Subcluster 4 was highly enriched in the MCAO group, and the metabolism herein showed elevation in amino acid metabolism, glycolysis, fatty acid metabolism, and gluconeogenesis [13]. However, to date, no study has investigated the heterogeneity and differential gene expressions between neurons in the infarct core and penumbra. Therefore, further investigations should address the aforementioned issue in an effect to provide potential targets for saving neurons in the ischemic penumbra.

## 7. Heterogeneity in endothelial cells in IS

Endothelial cells (ECs), vulnerable to ischemia, are a critical part of both the BBB and NVU [114]. Cerebral endothelium secretes vasodilators or vasoconstrictors, are involved in the communication network with other brain cells in an effect to regulate cerebral blood flow (CBF) and provide a non-thrombogenic surface [115, 116]. During IS, the main metabolic changes in ECs include the Golgi apparatus, gluconeogenesis and glycolysis, heme metabolism, and porphyrin. Endothelial dysfunction including oxidative stress damage and excess apoptosis induced by IS result in the aggravation of brain edema and neuroinflammation, impairment of endothelium-dependent vasodilation [117-119], reduction in CBF, along with an increased risk of intracerebral hemorrhage after thrombolytic therapy [120, 121]. Mechanistically, IS leads to dysfunction in BBB and hyperpermeability, thereby inducing damage of endothelial tight junctions, allowing water flow across the capillary, increase in transcytosis, and alteration in ion transport [122, 123]. Additionally, leukocyte adhesion molecules, VCAM-1 and ICAM-1, are significantly highly expressed post-stroke, thereby prompting leukocyte extravasation [117, 124]. Previous studies also showed that a possible mechanism underlying EC death post-stroke could be attributed to M1 microglia-derived TNF-α induced endothelial necroptosis and increased breakdown of BBB [125]. Additionally, anti-TNFα treatments, such as administration of infliximab, alleviate endothelial necroptosis and improve outcomes of stroke [126], suggesting a promising therapeutic modality. Nevertheless, the mechanisms underlying endothelial death and functional heterogeneity remain unclear and further investigations are needed to identify potential therapeutic targets for the treatment of injury in specific cell subtypes via scRNA-seq.

Using scRNA-seq, Guo et al. have divided ECs into 6 subclusters [14] (Table 1), and increased EC death induced by ischemia leads to a decrease in the cell number in the MCAO group, while BBB-related subclusters increase accompanied by the upregulation of BBB damage-associated genes, including Pdlim1, Timp1, Upp1, and Adamts4 [14]. The findings also demonstrate that IFN-I signaling genes, including Ifit3, Isg15, and Usp18, are highly expressed in two EC clusters, capillary (capEC-IFN^hi^) and arterial (aEC- IFN^lo^) [14]. Another scRNA-seq study employing GSVA reveals that cluster 3 varies significantly from all clusters and is the most enriched upon myogenesis and epithelial-mesenchymal transition [13]. GO and KEGG analyses show that cluster 3 is primarily oriented in the extracellular matrix, exosomes, and vasculature development, indicating the potential functions in extracellular communication and vascular reconstruction post-stroke [13]. Additionally, ECs in subcluster 3 show upregulated cyclic nucleotide metabolism, glutathione metabolism, ROS detoxification, and oxidative phosphorylation. Taken together, genes encoding inflammatory cytokines like Ccl4, Cd14, Cxcl2, and Spp1 are modulated by ischemia in each EC subcluster [13, 14], and are potential therapeutic targets.

## 8. Pericytic heterogeneity in IS revealed by scRNA-seq

Pericytes (PCs) are a significant component of the BBB as they maintain its normal physiological functioning [118]. PCs receive, modulate, and process signals to maintain normal homeostasis in the brain. They possess diverse neurovascular functions such as clearance of toxic metabolites, orchestration of BBB permeability, capillary hemodynamics, stem cell activity, and angiogenesis [127-129]. In recent years, accumulating evidence indicates that PCs may play vital roles in the pathogenesis of IS [130].

Due to their versatile functionality, some researchers speculate a heterogeneous cell population of PCs and each subcluster may play its specific role in IS [131]. A recent study has confirmed this hypothesis via scRNA-seq of PCs; 3 PCs subclusters have been identified (Table 1)[14]. PC0 subcluster is involved in the transport of calcium, sodium, and potassium ions, and maintaining normal functioning of the BBB [14]. Highly expressed gene sets in the PC1 subcluster are associated with inflammatory responses such as in cytokine and HIF-1-mediated signaling pathways [14]. The pathway involving syndecans 1 and muscle contraction associated with the multipotential differentiation capacity of PCs are enriched in the PC2 subcluster, accompanied by the specific upregulation of Acta2 in regulating the cerebral blood flow [14]. Although some initial progress has been made in evaluating the functional heterogeneity of various PC subpopulations by scRNA-seq, further studies are needed to develop genetic models and identify novel markers in distinct vessel segments combined with the findings of scRNA-seq and other novel technologies, thus providing novel and precise treatment for targeting specific genes expressed in the different PC subpopulations.

## 9. Vascular muscle smooth cells in IS

Vascular muscle smooth cells (VSMCs) are the major components of arteries and play a critical role in the modulation of blood pressure and CBF distribution in the CNS. As the main regulators of CBF, VSMCs modulate the relaxation and contraction of cerebral blood vessels [132, 133]. Cerebral ischemic injury may modify VSMC phenotypes, however, the underlying mechanisms remain unclear [133, 134], thus highlighting the importance of further studies for identifying potential therapeutic targets for future pharmacological development.

A recent study reports six VSMC subclusters (Table 1), along with the highly expressed genes associated with the specific markers in each subcluster in IS by scRNA-seq [14], including arteriole SMC subcluster0 (Tagln, Acta2), arterial SMC subclusters 1,2, and 3 (Eln, Ptgis, and Cnn1), and venous SMC subcluster5 (Art3, Car4) [14]. Notably, the activated SMC subcluster4 is specifically enriched in the processes of regulated exocytosis and neutrophil-mediated immunity, consistent with VSMC lysis and death which is driven by neutrophils in the case of atherosclerosis [14]. Additionally, subcluster5 includes the type I interferon signaling pathway that exhibits a reduced cell proportion in the MCAO group [14]. Further studies are required to evaluate the DEG-associated functions and gene regulatory network activities of each subcluster.

## 10. Heterogeneity in fibroblast-like cells in IS

Fibroblast-like cells (FBs) play significant roles in fibrosis, structural support, and injury repair of CNS [135-138]. Emerging evidence shows that FBs migrate to lesion sites post-stroke after the identification of PDGFRβ^+^ and CD105^+^ cells without PC markers, NG2 or CD13, but having higher fibronectin expression [139, 140]. However, the functions of FBs in ischemic are debatable. Col1α1^+^ and fibronectin^+^ FBs in peri-infarct regions highly express periostin-induced neural stem cell differentiation and proliferation as well as prompt neuro-restoration in the recovery phase of IS [141]. However, other studies report that the activation of PDGFRα expressed in FBs increases the cerebrovascular permeability in mice during IS [142, 143]. The dual roles in IS suggest that FBs exhibit functional heterogeneity post-stroke and various subclusters of FBs may possess different biological properties and functions during IS.

A scRNA-seq study reports three subclusters of FBs (Table 1)[14]. FB0 highly expresses genes associated with membrane transporters, including Slc1a3, Slc6a13, Slc7a11, and Slc22a6 as well as collagen fibril organizational genes, such as Col12a1, Col1a2, and Col1a1 [14]. FB1 is specifically enriched in the responses to IFN-β, including increased levels of Gsn, Ifitm2, and Ifitm1, and extracellular matrix organization, through Dcn, Lum, and Vcam1[14]. FB2 highly expresses genes associated with carboxylic acid transport, such as Slc6a6, Slc16a11, and Slc38a2, and those modulating intercellular pH, namely Slc4a10 and Slc26a2 [14]. Analysis of DEGs among different subclusters between the MCAO and sham groups indicates a subcluster-specific expression pattern for each subcluster [14]. The challenges herein can be attributed to the commonly shared numerous molecular markers between FB-like cells and PCs, making it difficult to differentiate them exactly. Further research is needed to identify specific markers in an effect to elucidate the functions of different FB subclusters in stroke.

## 11. Neutrophilic heterogeneity in IS

It is well acknowledged that neutrophils are detrimental factors after a stroke and efforts are underway to prevent neutrophils from entering the brain [144]. Neutrophils are reportedly the first immune cells that infiltrate into the ischemic area and provide vigorous and early inflammatory responses after IS [145]. Neutrophils are primarily recruited to the perivascular spaces in the ischemic area and target the NVU after a stroke [146]. In the early stages of stroke, neutrophils are activated and they attach to the endothelium [147] owing to the expression of endothelial adhesion molecules [148], including PSGL-1, MAC-1, CD11a, and ICAM-1. There is an evident increase in neutrophil number within a few hours after stroke [149]. During the acute stages, neutrophils aggravate IS-induced brain damage via several mechanisms [145]. For example, neutrophils physically block the microvascular network, thereby further decreasing CBF and contributing to the damage of adjacent tissues [150, 151]. Moreover, neutrophil count is directly related to the infarct size, and the neutrophil to lymphocyte ratio can be used to predict the risk of hemorrhagic transformation after IS [152, 153].

However, emerging evidence also suggests a possible neuroprotective role of neutrophils in IS [154]. Neutrophils exhibit functional heterogeneity and plasticity. They can transform into the N1 (pro-inflammatory phenotypes) or N2 (anti-inflammatory phenotypes) neutrophil types [155]. N1 or pro-inflammatory neutrophils induced by IFN-c possess a short life span and are associated with high cytotoxicity owing to the higher expression of TLR4, CD11b, CD86, and Ly6G [156]. In contrast, N2 or anti-inflammatory neutrophils are long-lived, characterized by the expression of CD206, YM-1, and Arg1 [157]. N2 neutrophils can be induced by TGFβ and produce anti-inflammatory cytokines thereby exerting neuroprotective effects and tissue remodeling [158]. A recent study reports the positive association between N2 neutrophils and the reduction of infarct size after stroke [154]. Therefore, the ratio of N1 to N2 may result in dissimilar clinical outcomes among stroke patients.

Notably, typing method of N1 and N2 oversimplifies the complicated phenotypic diversity of neutrophils [156]. Due to the advances in scRNA-seq, it is now possible to achieve a precise palette of the neutrophil population. Neutrophils are detrimental in the acute phases of IS but become neuroprotective at a later stage, suggesting reprogramming towards anti-inflammatory phenotypes, depending on activation signals and extracellular stimuli. Moreover, Cuartero et al. indicate that PPAR-γ pushes neutrophils toward a protective phenotype [154]. Interleukin-27 (IL-27) can also prompt neutrophilic reprogramming to anti-inflammatory phenotypes. Interestingly, scRNA-seq also demonstrates that bacterial infection may induce reprogramming of the neutrophilic population [159]. However, some studies suggest that neutrophils may be programmed before a stroke-induced insult [160-162], which may explain the distinct outcomes of stroke therapy.

A recent study reports four neutrophil subclusters via scRNA-seq [14]. The highly expressed genes and enriched pathways/processes are detailed in Table 4. Notably, there is a dynamic change in the compositional ratio of different neutrophil subpopulations at various stages of IS [14]. Further investigations are required to examine the relevant signals, the exact cell populations, and specific mechanisms involved in the reprogramming processes of neutrophils during IS by combining scRNA-seq with other new complementary technologies.

**Table 4:** Neutrophilic heterogeneity by scRNA-seq in IS.

Subpopulations	Highly expressed genes in IS	Enriched pathways and process	Refs.
**NEUT0**	Cxcl1, Hcar2, Ptafr, Cd63	Neutrophil degranulation, neutrophil activation involved in immune response, neutrophil mediated immunity, cellular response to interferon-gamma	[14]

**NEUT1**	Irf7, Isg15, Gbp2, Ifitm1	Type 1 interferon signaling pathway, cellular response to type 1 interferon, interferon-gamma-mediated signaling pathway, cellular response to interferon-gamma	[14]
**NEUT2**	Ccr1, Fpr1, Trem1, Ltb4r1, Cxcr2, Stfa2l1	Cellular response to cytokine stimulus, cytokine-mediated signaling pathway, inflammatory response, negative regulation of insulin receptor signaling and cellular response to insulin stimulus	[14]
**NEUT3**	Cebpe, Cd177, Cybb, Camp, Ltf	Granulocyte migration, defense response to fungus, innate immune response in mucosa, positive regulation of vesicle fusion, neutrophil extravasation	[14]

## 12. Lymphocytic heterogeneity in IS

### 12.1 T lymphocytes in IS

T lymphocytes can be subtyped as CD4^+^ T helper cells (Th) that modulate the functioning of granulocytes and phagocytes, CD8^+^ cytotoxic T lymphocytes (CTL), and regulatory T cells (Tregs) [163]. T lymphocytes can be activated and thus infiltrate into the ischemic brain and proliferate in the CNS as late as one week after IS [149]. Overall, it is well acknowledged that T lymphocytes amplify neuroinflammation and release cytotoxins and cytokines, thereby contributing to the progression to a secondary stroke [163]. However, emerging evidence shows that subpopulations of T lymphocytes exhibit tremendous heterogeneity in IS [164]. Previous studies suggest that mice deficient in Th1 and Th17 cells exhibit smaller infarct sizes and better recovery [165, 166]. The number of Th1 and Th17 cells increases within the first week after stroke, accompanied by an increase in the proinflammatory cytokines [167], including TNF-α, IL-1, IL-2, IL-12, and IL-17, thereby exacerbating the neurological damage. The mechanism underlying T cell activation in stroke is rather controversial, as some studies demonstrate that T cells are activated in stroke without an adaptive immune mechanism such as co-stimulatory pathways or antigen recognition [168], while others report that antigen-dependent activation of CTL can lead to the expansion of the infarct size [169]. Conversely, Th2 cells are considered to exert neuroprotective effects, along with the secretion of anti-inflammatory cytokines, including IL-13, IL-10, IL-5, and IL-4 in IS.

Growing evidence suggests that regulatory Tregs exhibit neuroprotective effects during IS rather than detrimental effects [170]. The proportion of Tregs, especially CD39^+^ Tregs, is greatly reduced during IS and is accompanied by the decrease in anti-inflammatory cytokines leading to the expansion of infarct sizes [170, 171]. Additionally, Tregs can limit neuroinflammation by inhibiting the MMP9 activity and maintaining the integrity of the BBB [172]. Moreover, MCAO mice with adoptive transfer of Tregs exhibit smaller infarct sizes and better outcomes within one week [173]. Nevertheless, selective depletion of Tregs can prompt neuro-restoration and microvascular dysfunction in an effect to decrease the infarct size [174].

A previous study indicates that mice with Treg depletion experience a rather slow recovery in motor functions, due to axonal myelination disorder in the external capsule and striatum after IS [10]. This suggests that Treg deficiency can impair the WM integrity and retard OL regeneration after IS. Interestingly, scRNA-seq analysis of Foxp3-expressing Tregs from the ischemic brain indicates that Tregs express genes to contact other T cells and microglial populations rather than directly contacting the OLs [10]. In PLX5622-treated mice with microglial depletion, newly matured OLs are not observed despite the adoptive transferring of exogenous Tregs after stroke, which further suggests that Tregs interact with microglia in an effect to affect oligodendrogenesis [10]. Mechanistically, Treg-derived osteopontin activates integrinβ1 receptors on the microglial surface, thereby increasing microglial protective functions, in turn resulting in OL maturation and WM repair post-stroke [10]. Shi et al. have identified a prominent Treg subcluster by scRNA-seq during the recovery stages of IS, thus providing a novel therapeutic method by increasing the effects of Tregs in the ischemic brain.

Collectively, despite the well-known deleterious roles of T lymphocytes in IS, evidence suggests the neuroprotective roles of Th2 and novel Tregs subclusters identified by scRNA-seq. Therefore, targeting the specific subclusters of T lymphocytes prompting neuroprotection is a promising therapeutic approach, however, further investigation is required to identify more optimal targets and the precise subclusters.

### 12.2 B lymphocytes and natural killer cells (NK) in IS

The production of local antibodies in CSF indicates the roles of B lymphocytes during IS [175]. The μMT knock-out mice, lacking mature B lymphocytes, exhibit a much larger infarct size and higher mortality post-stroke [176-178]. B lymphocytes produce IL-10 after IS, which can reduce the infiltration of neutrophils, macrophages, and T lymphocytes into the ischemic brain [177]. In 2020, Ortega et al.[179] showed that adoptively-transferred B lymphocytes reduce infarct sizes in the MCAO mice. Moreover, depletion of the B lymphocytes impairspatial memory, increase anxiety, and delay motor recovery, suggesting that diapedesis of B lymphocytes contributes to functional restoration after stroke. The beneficial effects of B lymphocytes are associated with the inhibition of cytotoxic immune cells. Nevertheless, other studies report the detrimental roles of B lymphocytes in IS. B lymphocyte infiltration into the ischemic area is sustained for at least 12 weeks post-stroke, resulting in cognitive decline, which suggests that B lymphocytes can be used to predict post-stroke dementia [180, 181]. Additionally, post-stroke cognitive decline is attenuated in B lymphocyte-deficient mice. Mechanistically, cognitive deficits induced by stroke may be associated with antibodies produced by B lymphocytes [181, 182]. In clinical trials, patients with post-stroke dementia showed a substantially higher level of B lymphocytes than those without dementia [181]. Therefore, after IS, B lymphocytes infiltrate the brain, and their functions vary at different stages of IS [181]. B lymphocytes secreting IL-10 result in a decrease in the infarct size and inflammatory responses at 48 hours post-stroke. However, B lymphocytes may induce post-stroke dementia during the chronic phases of IS [180, 183]. Furthermore, B lymphocytes also exhibit functional heterogeneity in IS; the B1 (Ly6d, Cd79a) and B2 subclusters (Ramp1, Lmo4) have been identified by scRNA-seq. However, the functions of the two different subclusters need further research in the future [14].

The roles of NK cells during IS are controversial [184]. NK cells can rapidly respond to ischemic insults by increasing cytotoxicity and inflammation, thereby exacerbating brain infarction [185, 186]. Mechanistically, NK cells release IFN-γ to recruit macrophages and dendritic cells, thus attenuating ischemic injury, while IFN-γ can also improve the survival of the infarcted brain by alleviating bacterial infections but not the lesion size [187]. A previous study divided NK cells in IS conditions into NK1 (Klrb1c, Ifng) and NK2 types (S100a1, Car2) by scRNA-seq analysis [14]. Previous studies indicate that the expression of TLR4 varies among different NK cell phenotypes and TLR4 increases the secretion of IFN-γ from the NK cells [188, 189]. However, more studies are needed to elucidate the role of TLR4 in different subclusters of NK cells during IS.

## 13. Conclusion and future perspectives

The emergence of scRNA-seq has allowed for targeting a particular cell type and thereby revealing the different phenotypic and molecular changes in cell types of interest during IS [13, 14]. This aids the detection of intercellular contact, cellular heterogeneity, and new treatments in stroke. Several cell types and subpopulations accompanied by the cell-type-specific gene expression patterns have been identified in IS penumbra, including glial cells, neurons, brain vasculature-associated cells, and peripheral immune cells. After describing the transcriptional landscape and “therapeutic time window” in stroke by scRNA-seq [13, 14], future studies should combine scRNA-seq and mechanistic examination to investigate the pathological process during the stroke for identifying new potential therapeutic targets.

**Figure 2.**
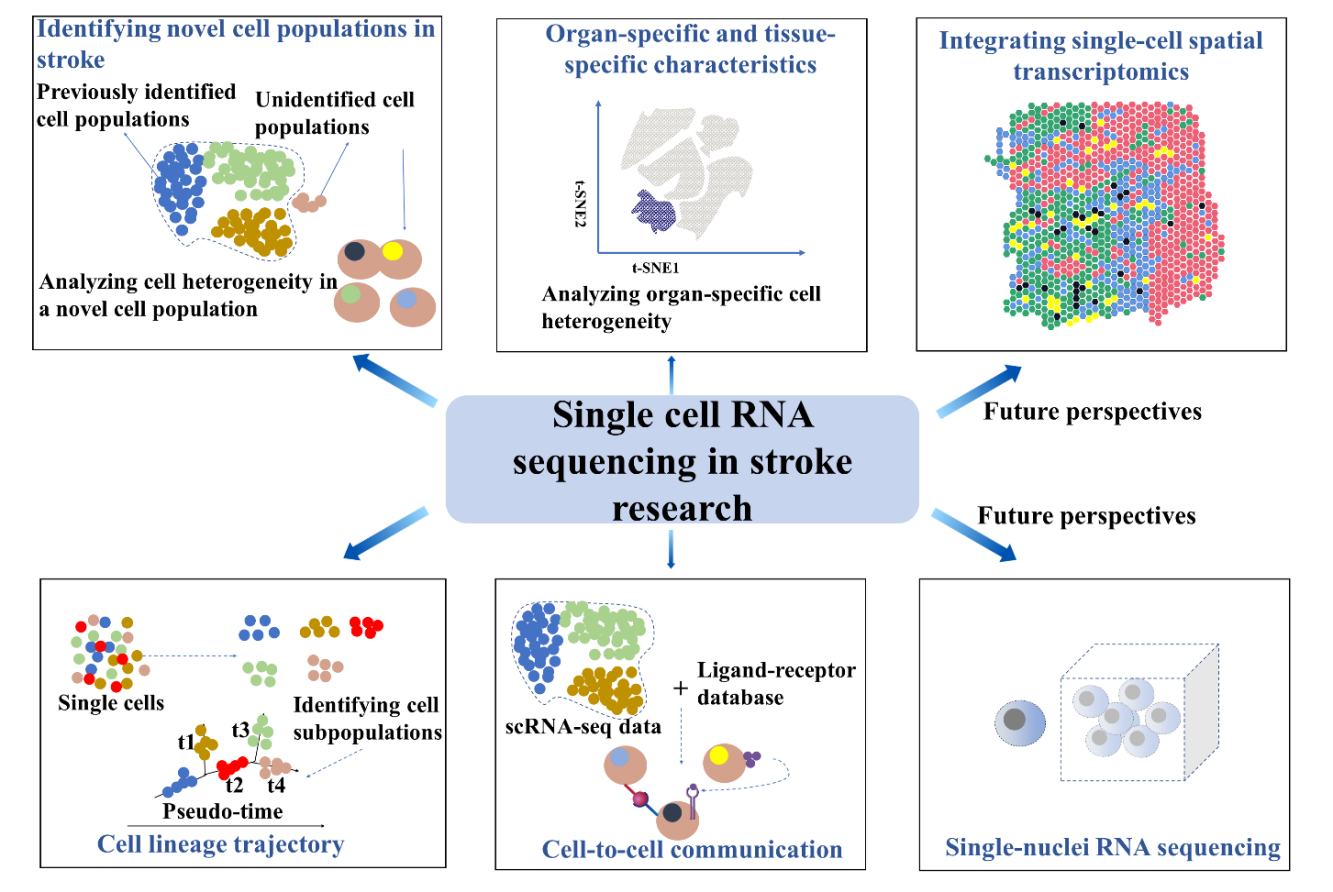
**Single-cell RNA sequencing in stroke research**. The advancements in scRNA-seq technologies present an unprecedented single-cell-resolution map of the brain. scRNA-seq is useful for identifying novel cell populations in stroke conditions and analyzing the cellular heterogeneity in a novel cell population. Additionally, scRNA-seq can reveal organ- or tissue-specific characteristics, cell lineage trajectories, as well as cell-to-cell communication states. Furthermore, single-nuclei RNA sequencing and single-cell spatial transcriptomics may provide a deeper understanding of the pathological mechanism during a stroke. Taken together, scRNA-seq contributes to the identification of potential new biomarkers, therapeutic targets, and the molecular underpinnings underlying pathological processes in stroke.

Due to the morphological complexity of cells in the CNS, many transcripts may be lost during sample preparation for scRNA-seq [190, 191]. Nuclear transcripts account for 20%-50% RNA in the cells, including unspliced and immature RNA molecules [192]. New methods such as single-nuclei RNA sequencing (snRNA-seq) are less influenced by technical artifacts during isolation unlike scRNA-seq [193, 194]. These reveal the localization of transcripts and provide different information for intronic versus exonic readers [195]; snRNA-seq may provide a deeper understanding of the pathological mechanisms during the stroke. scRNA-seq and snRNA-seq limit the spatial context, however, spatial connectivity and context between cells are significant to cell function [196, 197]. Single-cell spatial transcriptomics can combine the morphology of normal tissues, infarct core, and ischemic penumbra with sequencing data to yield more accurate information [198, 199] (Fig. 2).

Future studies combining scRNA-seq, snRNA-seq, single-cell spatial transcriptomics, and complimentary in-depth mechanistic investigations in stroke research will bring forth exciting findings in pathological structure-function changes, which may provide more effective treatment strategies for stroke therapy.

## References

[1] Campbell BCV, De Silva DA, Macleod MR, Coutts SB, Schwamm LH, Davis SM, et al. (2019). Ischaemic stroke. Nat Rev Dis Primers, 5:70. 31601801 10.1038/s41572-019-0118-8

[2] Prabhakaran S, Ruff I, Bernstein RA (2015). Acute stroke intervention: a systematic review. JAMA, 313:1451-1462. 25871671 10.1001/jama.2015.3058

[3] Herpich F, Rincon F (2020). Management of Acute Ischemic Stroke. Crit Care Med, 48:1654-1663. 32947473 10.1097/CCM.0000000000004597 PMC7540624

[4] Iadecola C (2017). The Neurovascular Unit Coming of Age: A Journey through Neurovascular Coupling in Health and Disease. Neuron, 96:17-42. 28957666 10.1016/j.neuron.2017.07.030 PMC5657612

[5] Arvanitis CD, Ferraro GB, Jain RK (2020). The blood-brain barrier and blood-tumour barrier in brain tumours and metastases. Nat Rev Cancer, 20:26-41. 31601988 10.1038/s41568-019-0205-x PMC8246629

[6] Jiang X, Andjelkovic AV, Zhu L, Yang T, Bennett MVL, Chen J, et al. (2018). Blood-brain barrier dysfunction and recovery after ischemic stroke. Prog Neurobiol, 163-164:144-171. 28987927 10.1016/j.pneurobio.2017.10.001 PMC5886838

[7] Sohrabji F, Bake S, Lewis DK (2013). Age-related changes in brain support cells: Implications for stroke severity. Neurochem Int, 63:291-301. 23811611 10.1016/j.neuint.2013.06.013 PMC3955169

[8] Jayaraj RL, Azimullah S, Beiram R, Jalal FY, Rosenberg GA (2019). Neuroinflammation: friend and foe for ischemic stroke. J Neuroinflammation, 16:142. 31291966 10.1186/s12974-019-1516-2 PMC6617684

[9] Morrison HW, Filosa JA (2019). Stroke and the neurovascular unit: glial cells, sex differences, and hypertension. Am J Physiol Cell Physiol, 316:C325-C339. 30601672 10.1152/ajpcell.00333.2018 PMC6457101

[10] Shi L, Sun Z, Su W, Xu F, Xie D, Zhang Q, et al. (2021). Treg cell-derived osteopontin promotes microglia-mediated white matter repair after ischemic stroke. Immunity, 54:1527-1542 e1528. 34015256 10.1016/j.immuni.2021.04.022 PMC8282725

[11] Shi X, Luo L, Wang J, Shen H, Li Y, Mamtilahun M, et al. (2021). Stroke subtype-dependent synapse elimination by reactive gliosis in mice. Nat Commun, 12:6943. 34836962 10.1038/s41467-021-27248-x PMC8626497

[12] Llorens-Bobadilla E, Zhao S, Baser A, Saiz-Castro G, Zwadlo K, Martin-Villalba A (2015). Single-Cell Transcriptomics Reveals a Population of Dormant Neural Stem Cells that Become Activated upon Brain Injury. Cell Stem Cell, 17:329-340. 26235341 10.1016/j.stem.2015.07.002

[13] Guo K, Luo J, Feng D, Wu L, Wang X, Xia L, et al. (2021). Single-Cell RNA Sequencing With Combined Use of Bulk RNA Sequencing to Reveal Cell Heterogeneity and Molecular Changes at Acute Stage of Ischemic Stroke in Mouse Cortex Penumbra Area. Front Cell Dev Biol, 9:624711. 33692998 10.3389/fcell.2021.624711 PMC7937629

[14] Zheng K, Lin L, Jiang W, Chen L, Zhang X, Zhang Q, et al. (2022). Single-cell RNA-seq reveals the transcriptional landscape in ischemic stroke. J Cereb Blood Flow Metab, 42:56-73. 34496660 10.1177/0271678X211026770 PMC8721774

[15] Butler A, Hoffman P, Smibert P, Papalexi E, Satija R (2018). Integrating single-cell transcriptomic data across different conditions, technologies, and species. Nat Biotechnol, 36:411-420. 29608179 10.1038/nbt.4096 PMC6700744

[16] Skinnider MA, Squair JW, Kathe C, Anderson MA, Gautier M, Matson KJE, et al. (2021). Cell type prioritization in single-cell data. Nat Biotechnol, 39:30-34. 32690972 10.1038/s41587-020-0605-1 PMC7610525

[17] Mu Q, Chen Y, Wang J (2019). Deciphering Brain Complexity Using Single-cell Sequencing. Genomics Proteomics Bioinformatics, 17:344-366. 31586689 10.1016/j.gpb.2018.07.007 PMC6943771

[18] Kulkarni A, Anderson AG, Merullo DP, Konopka G (2019). Beyond bulk: a review of single cell transcriptomics methodologies and applications. Curr Opin Biotechnol, 58:129-136. 30978643 10.1016/j.copbio.2019.03.001 PMC6710112

[19] DeSisto J, O'Rourke R, Jones HE, Pawlikowski B, Malek AD, Bonney S, et al. (2020). Single-Cell Transcriptomic Analyses of the Developing Meninges Reveal Meningeal Fibroblast Diversity and Function. Dev Cell, 54:43-59 e44. 32634398 10.1016/j.devcel.2020.06.009 PMC7769050

[20] Corley MJ, Farhadian SF (2021). Emerging Single-cell Approaches to Understand HIV in the Central Nervous System. Curr HIV/AIDS Rep. 10.1007/s11904-021-00586-7 PMC8613726 34822063

[21] Gaublomme JT, Yosef N, Lee Y, Gertner RS, Yang LV, Wu C, et al. (2015). Single-Cell Genomics Unveils Critical Regulators of Th17 Cell Pathogenicity. Cell, 163:1400-1412. 26607794 10.1016/j.cell.2015.11.009 PMC4671824

[22] Sabbagh MF, Heng JS, Luo C, Castanon RG, Nery JR, Rattner A, et al. (2018). Transcriptional and epigenomic landscapes of CNS and non-CNS vascular endothelial cells. Elife, 7. 10.7554/eLife.36187 PMC6126923 30188322

[23] Travaglini KJ, Nabhan AN, Penland L, Sinha R, Gillich A, Sit RV, et al. (2020). A molecular cell atlas of the human lung from single-cell RNA sequencing. Nature, 587:619-625. 33208946 10.1038/s41586-020-2922-4 PMC7704697

[24] Denisenko E, Guo BB, Jones M, Hou R, de Kock L, Lassmann T, et al. (2020). Systematic assessment of tissue dissociation and storage biases in single-cell and single-nucleus RNA-seq workflows. Genome Biol, 21:130. 32487174 10.1186/s13059-020-02048-6 PMC7265231

[25] Kiss T, Nyul-Toth A, Balasubramanian P, Tarantini S, Ahire C, DelFavero J, et al. (2020). Single-cell RNA sequencing identifies senescent cerebromicrovascular endothelial cells in the aged mouse brain. Geroscience, 42:429-444. 32236824 10.1007/s11357-020-00177-1 PMC7205992

[26] Feyen DAM, Perea-Gil I, Maas RGC, Harakalova M, Gavidia AA, Arthur Ataam J, et al. (2021). Unfolded Protein Response as a Compensatory Mechanism and Potential Therapeutic Target in PLN R14del Cardiomyopathy. Circulation, 144:382-392. 33928785 10.1161/CIRCULATIONAHA.120.049844 PMC8667423

[27] Paik DT, Tian L, Lee J, Sayed N, Chen IY, Rhee S, et al. (2018). Large-Scale Single-Cell RNA-Seq Reveals Molecular Signatures of Heterogeneous Populations of Human Induced Pluripotent Stem Cell-Derived Endothelial Cells. Circ Res, 123:443-450. 29986945 10.1161/CIRCRESAHA.118.312913 PMC6202208

[28] Bradshaw EM, Chibnik LB, Keenan BT, Ottoboni L, Raj T, Tang A, et al. (2013). CD33 Alzheimer's disease locus: altered monocyte function and amyloid biology. Nat Neurosci, 16:848-850. 23708142 10.1038/nn.3435 PMC3703870

[29] Jay TR, Miller CM, Cheng PJ, Graham LC, Bemiller S, Broihier ML, et al. (2015). TREM2 deficiency eliminates TREM2+ inflammatory macrophages and ameliorates pathology in Alzheimer's disease mouse models. J Exp Med, 212:287-295. 25732305 10.1084/jem.20142322 PMC4354365

[30] Ransohoff RM (2016). How neuroinflammation contributes to neurodegeneration. Science, 353:777-783. 27540165 10.1126/science.aag2590

[31] Chan G, White CC, Winn PA, Cimpean M, Replogle JM, Glick LR, et al. (2016). Trans-pQTL study identifies immune crosstalk between Parkinson and Alzheimer loci. Neurol Genet, 2:e90. 27504496 10.1212/NXG.0000000000000090 PMC4962525

[32] Jaitin DA, Weiner A, Yofe I, Lara-Astiaso D, Keren-Shaul H, David E, et al. (2016). Dissecting Immune Circuits by Linking CRISPR-Pooled Screens with Single-Cell RNA-Seq. Cell, 167:1883-1896 e1815. 27984734 10.1016/j.cell.2016.11.039

[33] Ofengeim D, Giagtzoglou N, Huh D, Zou C, Yuan J (2017). Single-Cell RNA Sequencing: Unraveling the Brain One Cell at a Time. Trends Mol Med, 23:563-576. 28501348 10.1016/j.molmed.2017.04.006 PMC5531055

[34] Colonna M, Butovsky O (2017). Microglia Function in the Central Nervous System During Health and Neurodegeneration. Annu Rev Immunol, 35:441-468. 28226226 10.1146/annurev-immunol-051116-052358 PMC8167938

[35] McKinsey GL, Lizama CO, Keown-Lang AE, Niu A, Santander N, Larpthaveesarp A, et al. (2020). A new genetic strategy for targeting microglia in development and disease. Elife, 9. 10.7554/eLife.54590 PMC7375817 32573436

[36] Bottcher C, Schlickeiser S, Sneeboer MAM, Kunkel D, Knop A, Paza E, et al. (2019). Human microglia regional heterogeneity and phenotypes determined by multiplexed single-cell mass cytometry. Nat Neurosci, 22:78-90. 30559476 10.1038/s41593-018-0290-2

[37] Li Q, Cheng Z, Zhou L, Darmanis S, Neff NF, Okamoto J, et al. (2019). Developmental Heterogeneity of Microglia and Brain Myeloid Cells Revealed by Deep Single-Cell RNA Sequencing. Neuron, 101:207-223 e210. 30606613 10.1016/j.neuron.2018.12.006 PMC6336504

[38] Tan YL, Yuan Y, Tian L (2020). Microglial regional heterogeneity and its role in the brain. Mol Psychiatry, 25:351-367. 31772305 10.1038/s41380-019-0609-8 PMC6974435

[39] Nayak D, Roth TL, McGavern DB (2014). Microglia development and function. Annu Rev Immunol, 32:367-402. 24471431 10.1146/annurev-immunol-032713-120240 PMC5001846

[40] Wright-Jin EC, Gutmann DH (2019). Microglia as Dynamic Cellular Mediators of Brain Function. Trends Mol Med, 25:967-979. 31597593 10.1016/j.molmed.2019.08.013 PMC6829057

[41] Sankowski R, Bottcher C, Masuda T, Geirsdottir L, Sagar, Sindram E, et al. (2019). Mapping microglia states in the human brain through the integration of high-dimensional techniques. Nat Neurosci, 22:2098-2110. 31740814 10.1038/s41593-019-0532-y

[42] Tang Y, Le W (2016). Differential Roles of M1 and M2 Microglia in Neurodegenerative Diseases. Mol Neurobiol, 53:1181-1194. 25598354 10.1007/s12035-014-9070-5

[43] Wolf SA, Boddeke HW, Kettenmann H (2017). Microglia in Physiology and Disease. Annu Rev Physiol, 79:619-643. 27959620 10.1146/annurev-physiol-022516-034406

[44] Ochocka N, Kaminska B (2021). Microglia Diversity in Healthy and Diseased Brain: Insights from Single-Cell Omics. Int J Mol Sci, 22. 33809675 10.3390/ijms22063027 PMC8002227

[45] Qin C, Zhou LQ, Ma XT, Hu ZW, Yang S, Chen M, et al. (2019). Dual Functions of Microglia in Ischemic Stroke. Neurosci Bull, 35:921-933. 31062335 10.1007/s12264-019-00388-3 PMC6754485

[46] Ma Y, Wang J, Wang Y, Yang GY (2017). The biphasic function of microglia in ischemic stroke. Prog Neurobiol, 157:247-272. 26851161 10.1016/j.pneurobio.2016.01.005

[47] Kronenberg G, Uhlemann R, Richter N, Klempin F, Wegner S, Staerck L, et al. (2018). Distinguishing features of microglia- and monocyte-derived macrophages after stroke. Acta Neuropathol, 135:551-568. 29249001 10.1007/s00401-017-1795-6

[48] Kanazawa M, Ninomiya I, Hatakeyama M, Takahashi T, Shimohata T (2017). Microglia and Monocytes/Macrophages Polarization Reveal Novel Therapeutic Mechanism against Stroke. Int J Mol Sci, 18. 10.3390/ijms18102135 PMC5666817 29027964

[49] Otxoa-de-Amezaga A, Miro-Mur F, Pedragosa J, Gallizioli M, Justicia C, Gaja-Capdevila N, et al. (2019). Microglial cell loss after ischemic stroke favors brain neutrophil accumulation. Acta Neuropathol, 137:321-341. 30580383 10.1007/s00401-018-1954-4 PMC6513908

[50] Masuda T, Sankowski R, Staszewski O, Prinz M (2020). Microglia Heterogeneity in the Single-Cell Era. Cell Rep, 30:1271-1281. 32023447 10.1016/j.celrep.2020.01.010

[51] Singhal G, Baune BT (2017). Microglia: An Interface between the Loss of Neuroplasticity and Depression. Front Cell Neurosci, 11:270. 28943841 10.3389/fncel.2017.00270 PMC5596091

[52] Sun J, Pan X, Christiansen LI, Yuan XL, Skovgaard K, Chatterton DEW, et al. (2018). Necrotizing enterocolitis is associated with acute brain responses in preterm pigs. J Neuroinflammation, 15:180. 29885660 10.1186/s12974-018-1201-x PMC5994241

[53] Xu Z, Ford GD, Croslan DR, Jiang J, Gates A, Allen R, et al. (2005). Neuroprotection by neuregulin-1 following focal stroke is associated with the attenuation of ischemia-induced pro-inflammatory and stress gene expression. Neurobiol Dis, 19:461-470. 16023588 10.1016/j.nbd.2005.01.027

[54] Chelluboina B, Klopfenstein JD, Pinson DM, Wang DZ, Vemuganti R, Veeravalli KK (2015). Matrix Metalloproteinase-12 Induces Blood-Brain Barrier Damage After Focal Cerebral Ischemia. Stroke, 46:3523-3531. 26534974 10.1161/STROKEAHA.115.011031

[55] Schlomann U, Rathke-Hartlieb S, Yamamoto S, Jockusch H, Bartsch JW (2000). Tumor necrosis factor alpha induces a metalloprotease-disintegrin, ADAM8 (CD 156): implications for neuron-glia interactions during neurodegeneration. J Neurosci, 20:7964-7971. 11050116 10.1523/JNEUROSCI.20-21-07964.2000 PMC6772711

[56] Aibar S, Gonzalez-Blas CB, Moerman T, Huynh-Thu VA, Imrichova H, Hulselmans G, et al. (2017). SCENIC: single-cell regulatory network inference and clustering. Nat Methods, 14:1083-1086. 28991892 10.1038/nmeth.4463 PMC5937676

[57] Zhang YY, Wang K, Liu YE, Wang W, Liu AF, Zhou J, et al. (2019). Identification of key transcription factors associated with cerebral ischemiareperfusion injury based on geneset enrichment analysis. Int J Mol Med, 43:2429-2439. 31017267 10.3892/ijmm.2019.4159 PMC6488172

[58] Kozela E, Juknat A, Gao F, Kaushansky N, Coppola G, Vogel Z (2016). Pathways and gene networks mediating the regulatory effects of cannabidiol, a nonpsychoactive cannabinoid, in autoimmune T cells. J Neuroinflammation, 13:136. 27256343 10.1186/s12974-016-0603-x PMC4891926

[59] Formisano L, Guida N, Valsecchi V, Cantile M, Cuomo O, Vinciguerra A, et al. (2015). Sp3/REST/HDAC1/HDAC2 Complex Represses and Sp1/HIF-1/p300 Complex Activates ncx1 Gene Transcription, in Brain Ischemia and in Ischemic Brain Preconditioning, by Epigenetic Mechanism. J Neurosci, 35:7332-7348. 25972164 10.1523/JNEUROSCI.2174-14.2015 PMC6705442

[60] Wu SC, Zhang Y (2011). Cyclin-dependent kinase 1 (CDK1)-mediated phosphorylation of enhancer of zeste 2 (Ezh2) regulates its stability. J Biol Chem, 286:28511-28519. 21659531 10.1074/jbc.M111.240515 PMC3151093

[61] Liu B, Zhang YH, Jiang Y, Li LL, Chen Q, He GQ, et al. (2015). Gadd45b is a novel mediator of neuronal apoptosis in ischemic stroke. Int J Biol Sci, 11:353-360. 25678854 10.7150/ijbs.9813 PMC4323375

[62] Diao J, Zhang C, Zhang D, Wang X, Zhang J, Ma C, et al. (2019). Role and mechanisms of a three-dimensional bioprinted microtissue model in promoting proliferation and invasion of growth-hormone-secreting pituitary adenoma cells. Biofabrication, 11:025006. 30537696 10.1088/1758-5090/aaf7ea

[63] Collmann FM, Pijnenburg R, Hamzei-Taj S, Minassian A, Folz-Donahue K, Kukat C, et al. (2019). Individual in vivo Profiles of Microglia Polarization After Stroke, Represented by the Genes iNOS and Ym1. Front Immunol, 10:1236. 31214190 10.3389/fimmu.2019.01236 PMC6558167

[64] Kobashi S, Terashima T, Katagi M, Nakae Y, Okano J, Suzuki Y, et al. (2020). Transplantation of M2-Deviated Microglia Promotes Recovery of Motor Function after Spinal Cord Injury in Mice. Mol Ther, 28:254-265. 31604678 10.1016/j.ymthe.2019.09.004 PMC6952178

[65] Alam MA, Subramanyam Rallabandi VP, Roy PK (2016). Systems Biology of Immunomodulation for Post-Stroke Neuroplasticity: Multimodal Implications of Pharmacotherapy and Neurorehabilitation. Front Neurol, 7:94. 27445961 10.3389/fneur.2016.00094 PMC4923163

[66] He Y, Ma X, Li D, Hao J (2017). Thiamet G mediates neuroprotection in experimental stroke by modulating microglia/macrophage polarization and inhibiting NF-kappaB p65 signaling. J Cereb Blood Flow Metab, 37:2938-2951. 27864466 10.1177/0271678X16679671 PMC5536801

[67] Orihuela R, McPherson CA, Harry GJ (2016). Microglial M1/M2 polarization and metabolic states. Br J Pharmacol, 173:649-665. 25800044 10.1111/bph.13139 PMC4742299

[68] Liddelow SA, Barres BA (2017). Reactive Astrocytes: Production, Function, and Therapeutic Potential. Immunity, 46:957-967. 28636962 10.1016/j.immuni.2017.06.006

[69] Perea G, Navarrete M, Araque A (2009). Tripartite synapses: astrocytes process and control synaptic information. Trends Neurosci, 32:421-431. 19615761 10.1016/j.tins.2009.05.001

[70] Theparambil SM, Hosford PS, Ruminot I, Kopach O, Reynolds JR, Sandoval PY, et al. (2020). Astrocytes regulate brain extracellular pH via a neuronal activity-dependent bicarbonate shuttle. Nat Commun, 11:5073. 33033238 10.1038/s41467-020-18756-3 PMC7545092

[71] Sofroniew MV (2020). Astrocyte Reactivity: Subtypes, States, and Functions in CNS Innate Immunity. Trends Immunol, 41:758-770. 32819810 10.1016/j.it.2020.07.004 PMC7484257

[72] Pekny M, Nilsson M (2005). Astrocyte activation and reactive gliosis. Glia, 50:427-434. 15846805 10.1002/glia.20207

[73] Vecino E, Rodriguez FD, Ruzafa N, Pereiro X, Sharma SC (2016). Glia-neuron interactions in the mammalian retina. Prog Retin Eye Res, 51:1-40. 26113209 10.1016/j.preteyeres.2015.06.003

[74] Duran J, Guinovart JJ (2015). Brain glycogen in health and disease. Mol Aspects Med, 46:70-77. 26344371 10.1016/j.mam.2015.08.007

[75] Guo H, Fan Z, Wang S, Ma L, Wang J, Yu D, et al. (2021). Astrocytic A1/A2 paradigm participates in glycogen mobilization mediated neuroprotection on reperfusion injury after ischemic stroke. J Neuroinflammation, 18:230. 34645472 10.1186/s12974-021-02284-y PMC8513339

[76] Zhao N, Xu X, Jiang Y, Gao J, Wang F, Xu X, et al. (2019). Lipocalin-2 may produce damaging effect after cerebral ischemia by inducing astrocytes classical activation. J Neuroinflammation, 16:168. 31426811 10.1186/s12974-019-1556-7 PMC6699078

[77] Okada S, Nakamura M, Katoh H, Miyao T, Shimazaki T, Ishii K, et al. (2006). Conditional ablation of Stat3 or Socs3 discloses a dual role for reactive astrocytes after spinal cord injury. Nat Med, 12:829-834. 16783372 10.1038/nm1425

[78] Aswendt M, Wilhelmsson U, Wieters F, Stokowska A, Schmitt FJ, Pallast N, et al. (2021). Reactive Astrocytes Prevent Maladaptive Plasticity after Ischemic Stroke. Prog Neurobiol:102199. 34921928 10.1016/j.pneurobio.2021.102199

[79] Barca C, Wiesmann M, Calahorra J, Wachsmuth L, Doring C, Foray C, et al. (2021). Impact of hydroxytyrosol on stroke: tracking therapy response on neuroinflammation and cerebrovascular parameters using PET-MR imaging and on functional outcomes. Theranostics, 11:4030-4049. 33754046 10.7150/thno.48110 PMC7977466

[80] Becchi S, Buson A, Balleine BW (2021). Inhibition of vascular adhesion protein 1 protects dopamine neurons from the effects of acute inflammation and restores habit learning in the striatum. J Neuroinflammation, 18:233. 34654450 10.1186/s12974-021-02288-8 PMC8520223

[81] Liang Z, Wang X, Hao Y, Qiu L, Lou Y, Zhang Y, et al. (2020). The Multifaceted Role of Astrocyte Connexin 43 in Ischemic Stroke Through Forming Hemichannels and Gap Junctions. Front Neurol, 11:703. 32849190 10.3389/fneur.2020.00703 PMC7411525

[82] Hayes CA, Ashmore BG, Vijayasankar A, Marshall JP, Ashpole NM (2021). Insulin-Like Growth Factor-1 Differentially Modulates Glutamate-Induced Toxicity and Stress in Cells of the Neurogliovascular Unit. Front Aging Neurosci, 13:751304. 34887742 10.3389/fnagi.2021.751304 PMC8650493

[83] Shen XY, Gao ZK, Han Y, Yuan M, Guo YS, Bi X (2021). Activation and Role of Astrocytes in Ischemic Stroke. Front Cell Neurosci, 15:755955. 34867201 10.3389/fncel.2021.755955 PMC8635513

[84] Zhang D, Zhao S, Zhang Z, Xu D, Lian D, Wu J, et al. (2021). Regulation of the p75 neurotrophin receptor attenuates neuroinflammation and stimulates hippocampal neurogenesis in experimental Streptococcus pneumoniae meningitis. J Neuroinflammation, 18:253. 34727939 10.1186/s12974-021-02294-w PMC8561879

[85] Wang X, Chen S, Ni J, Cheng J, Jia J, Zhen X (2018). miRNA-3473b contributes to neuroinflammation following cerebral ischemia. Cell Death Dis, 9:11. 29317607 10.1038/s41419-017-0014-7 PMC5849032

[86] Fasolo F, Jin H, Winski G, Chernogubova E, Pauli J, Winter H, et al. (2021). Long Noncoding RNA MIAT Controls Advanced Atherosclerotic Lesion Formation and Plaque Destabilization. Circulation, 144:1567-1583. 34647815 10.1161/CIRCULATIONAHA.120.052023 PMC8570347

[87] Chung WS, Clarke LE, Wang GX, Stafford BK, Sher A, Chakraborty C, et al. (2013). Astrocytes mediate synapse elimination through MEGF10 and MERTK pathways. Nature, 504:394-400. 24270812 10.1038/nature12776 PMC3969024

[88] Damisah EC, Hill RA, Rai A, Chen F, Rothlin CV, Ghosh S, et al. (2020). Astrocytes and microglia play orchestrated roles and respect phagocytic territories during neuronal corpse removal in vivo. Sci Adv, 6:eaba3239. 32637606 10.1126/sciadv.aba3239 PMC7319765

[89] Greenhalgh AD, David S, Bennett FC (2020). Immune cell regulation of glia during CNS injury and disease. Nat Rev Neurosci, 21:139-152. 32042145 10.1038/s41583-020-0263-9

[90] Stadelmann C, Timmler S, Barrantes-Freer A, Simons M (2019). Myelin in the Central Nervous System: Structure, Function, and Pathology. Physiol Rev, 99:1381-1431. 31066630 10.1152/physrev.00031.2018

[91] Huang W, Bhaduri A, Velmeshev D, Wang S, Wang L, Rottkamp CA, et al. (2020). Origins and Proliferative States of Human Oligodendrocyte Precursor Cells. Cell, 182:594-608 e511. 32679030 10.1016/j.cell.2020.06.027 PMC7415734

[92] Kirby L, Jin J, Cardona JG, Smith MD, Martin KA, Wang J, et al. (2019). Oligodendrocyte precursor cells present antigen and are cytotoxic targets in inflammatory demyelination. Nat Commun, 10:3887. 31467299 10.1038/s41467-019-11638-3 PMC6715717

[93] Petersen MA, Ryu JK, Chang KJ, Etxeberria A, Bardehle S, Mendiola AS, et al. (2017). Fibrinogen Activates BMP Signaling in Oligodendrocyte Progenitor Cells and Inhibits Remyelination after Vascular Damage. Neuron, 96:1003-1012 e1007. 29103804 10.1016/j.neuron.2017.10.008 PMC5851281

[94] Philips T, Rothstein JD (2017). Oligodendroglia: metabolic supporters of neurons. J Clin Invest, 127:3271-3280. 28862639 10.1172/JCI90610 PMC5669561

[95] Saab AS, Tzvetanova ID, Nave KA (2013). The role of myelin and oligodendrocytes in axonal energy metabolism. Curr Opin Neurobiol, 23:1065-1072. 24094633 10.1016/j.conb.2013.09.008

[96] Xu S, Lu J, Shao A, Zhang JH, Zhang J (2020). Glial Cells: Role of the Immune Response in Ischemic Stroke. Front Immunol, 11:294. 32174916 10.3389/fimmu.2020.00294 PMC7055422

[97] Liu S, Jin R, Xiao AY, Zhong W, Li G (2019). Inhibition of CD147 improves oligodendrogenesis and promotes white matter integrity and functional recovery in mice after ischemic stroke. Brain Behav Immun, 82:13-24. 31356925 10.1016/j.bbi.2019.07.027 PMC6800638

[98] Bacmeister CM, Barr HJ, McClain CR, Thornton MA, Nettles D, Welle CG, et al. (2020). Motor learning promotes remyelination via new and surviving oligodendrocytes. Nat Neurosci, 23:819-831. 32424285 10.1038/s41593-020-0637-3 PMC7329620

[99] Garcia-Martin G, Alcover-Sanchez B, Wandosell F, Cubelos B (2021). Pathways involved in remyelination after cerebral ischemia. Curr Neuropharmacol. 10.2174/1570159X19666210610093658 PMC9878953 34151767

[100] Kishida N, Maki T, Takagi Y, Yasuda K, Kinoshita H, Ayaki T, et al. (2019). Role of Perivascular Oligodendrocyte Precursor Cells in Angiogenesis After Brain Ischemia. J Am Heart Assoc, 8:e011824. 31020902 10.1161/JAHA.118.011824 PMC6512138

[101] Tirotta E, Ransohoff RM, Lane TE (2011). CXCR2 signaling protects oligodendrocyte progenitor cells from IFN-gamma/CXCL10-mediated apoptosis. Glia, 59:1518-1528. 21656856 10.1002/glia.21195 PMC5029281

[102] Valentin-Torres A, Savarin C, Barnett J, Bergmann CC (2018). Blockade of sustained tumor necrosis factor in a transgenic model of progressive autoimmune encephalomyelitis limits oligodendrocyte apoptosis and promotes oligodendrocyte maturation. J Neuroinflammation, 15:121. 29690885 10.1186/s12974-018-1164-y PMC5916830

[103] Liu H, Yang X, Yang J, Yuan Y, Wang Y, Zhang R, et al. (2021). IL-17 Inhibits Oligodendrocyte Progenitor Cell Proliferation and Differentiation by Increasing K(+) Channel Kv1.3. Front Cell Neurosci, 15:679413. 34239419 10.3389/fncel.2021.679413 PMC8258110

[104] Li X, Qin L, Li Y, Yu H, Zhang Z, Tao C, et al. (2019). Presynaptic Endosomal Cathepsin D Regulates the Biogenesis of GABAergic Synaptic Vesicles. Cell Rep, 28:1015-1028.e1015. 31340140 10.1016/j.celrep.2019.06.006

[105] Bonetto G, Belin D, Karadottir RT (2021). Myelin: A gatekeeper of activity-dependent circuit plasticity? Science, 374:eaba6905. 34618550 10.1126/science.aba6905

[106] Schinder AF, Olson EC, Spitzer NC, Montal M (1996). Mitochondrial dysfunction is a primary event in glutamate neurotoxicity. J Neurosci, 16:6125-6133. 8815895 10.1523/JNEUROSCI.16-19-06125.1996 PMC6579180

[107] Morizawa YM, Hirayama Y, Ohno N, Shibata S, Shigetomi E, Sui Y, et al. (2017). Reactive astrocytes function as phagocytes after brain ischemia via ABCA1-mediated pathway. Nat Commun, 8:28. 28642575 10.1038/s41467-017-00037-1 PMC5481424

[108] Deleglise B, Lassus B, Soubeyre V, Doulazmi M, Brugg B, Vanhoutte P, et al. (2018). Dysregulated Neurotransmission induces Trans-synaptic degeneration in reconstructed Neuronal Networks. Sci Rep, 8:11596. 30072750 10.1038/s41598-018-29918-1 PMC6072786

[109] Terasaki Y, Liu Y, Hayakawa K, Pham LD, Lo EH, Ji X, et al. (2014). Mechanisms of neurovascular dysfunction in acute ischemic brain. Curr Med Chem, 21:2035-2042. 24372202 10.2174/0929867321666131228223400 PMC4066327

[110] Harrison EB, Hochfelder CG, Lamberty BG, Meays BM, Morsey BM, Kelso ML, et al. (2016). Traumatic brain injury increases levels of miR-21 in extracellular vesicles: implications for neuroinflammation. FEBS Open Bio, 6:835-846. 10.1002/2211-5463.12092 PMC4971839 27516962

[111] Agnati LF, Fuxe K (2014). Extracellular-vesicle type of volume transmission and tunnelling-nanotube type of wiring transmission add a new dimension to brain neuro-glial networks. Philos Trans R Soc Lond B Biol Sci, 369. 10.1098/rstb.2013.0505 PMC4142026 25135966

[112] Jones EV, Bernardinelli Y, Zarruk JG, Chierzi S, Murai KK (2018). SPARC and GluA1-Containing AMPA Receptors Promote Neuronal Health Following CNS Injury. Front Cell Neurosci, 12:22. 29449802 10.3389/fncel.2018.00022 PMC5799273

[113] Budnik V, Ruiz-Canada C, Wendler F (2016). Extracellular vesicles round off communication in the nervous system. Nat Rev Neurosci, 17:160-172. 26891626 10.1038/nrn.2015.29 PMC4989863

[114] Daneman R, Prat A (2015). The blood-brain barrier. Cold Spring Harb Perspect Biol, 7:a020412. 25561720 10.1101/cshperspect.a020412 PMC4292164

[115] Yang C, Hawkins KE, Dore S, Candelario-Jalil E (2019). Neuroinflammatory mechanisms of blood-brain barrier damage in ischemic stroke. Am J Physiol Cell Physiol, 316:C135-C153. 30379577 10.1152/ajpcell.00136.2018 PMC6397344

[116] Hu X, De Silva TM, Chen J, Faraci FM (2017). Cerebral Vascular Disease and Neurovascular Injury in Ischemic Stroke. Circ Res, 120:449-471. 28154097 10.1161/CIRCRESAHA.116.308427 PMC5313039

[117] Zhou YF, Chen AQ, Wu JH, Mao L, Xia YP, Jin HJ, et al. (2019). Sema3E/PlexinD1 signaling inhibits postischemic angiogenesis by regulating endothelial DLL4 and filopodia formation in a rat model of ischemic stroke. FASEB J, 33:4947-4961. 30653356 10.1096/fj.201801706RR

[118] Zhou YF, Li PC, Wu JH, Haslam JA, Mao L, Xia YP, et al. (2018). Sema3E/PlexinD1 inhibition is a therapeutic strategy for improving cerebral perfusion and restoring functional loss after stroke in aged rats. Neurobiol Aging, 70:102-116. 30007159 10.1016/j.neurobiolaging.2018.06.003

[119] Wu JH, Li YN, Chen AQ, Hong CD, Zhang CL, Wang HL, et al. (2020). Inhibition of Sema4D/PlexinB1 signaling alleviates vascular dysfunction in diabetic retinopathy. EMBO Mol Med, 12:e10154. 31943789 10.15252/emmm.201810154 PMC7005627

[120] Petersen MA, Ryu JK, Akassoglou K (2018). Fibrinogen in neurological diseases: mechanisms, imaging and therapeutics. Nat Rev Neurosci, 19:283-301. 29618808 10.1038/nrn.2018.13 PMC6743980

[121] Shi Y, Jiang X, Zhang L, Pu H, Hu X, Zhang W, et al. (2017). Endothelium-targeted overexpression of heat shock protein 27 ameliorates blood-brain barrier disruption after ischemic brain injury. Proc Natl Acad Sci U S A, 114:E1243-E1252. 28137866 10.1073/pnas.1621174114 PMC5320958

[122] Sladojevic N, Stamatovic SM, Johnson AM, Choi J, Hu A, Dithmer S, et al. (2019). Claudin-1-Dependent Destabilization of the Blood-Brain Barrier in Chronic Stroke. J Neurosci, 39:743-757. 30504279 10.1523/JNEUROSCI.1432-18.2018 PMC6343646

[123] Nag S, Manias JL, Stewart DJ (2009). Expression of endothelial phosphorylated caveolin-1 is increased in brain injury. Neuropathol Appl Neurobiol, 35:417-426. 19508446 10.1111/j.1365-2990.2008.01009.x

[124] Whiteley WN, Emberson J, Lees KR, Blackwell L, Albers G, Bluhmki E, et al. (2016). Risk of intracerebral haemorrhage with alteplase after acute ischaemic stroke: a secondary analysis of an individual patient data meta-analysis. Lancet Neurol, 15:925-933. 27289487 10.1016/S1474-4422(16)30076-X

[125] Pan J, Jin JL, Ge HM, Yin KL, Chen X, Han LJ, et al. (2015). Malibatol A regulates microglia M1/M2 polarization in experimental stroke in a PPARgamma-dependent manner. J Neuroinflammation, 12:51. 25889216 10.1186/s12974-015-0270-3 PMC4378556

[126] Chen AQ, Fang Z, Chen XL, Yang S, Zhou YF, Mao L, et al. (2019). Microglia-derived TNF-alpha mediates endothelial necroptosis aggravating blood brain-barrier disruption after ischemic stroke. Cell Death Dis, 10:487. 31221990 10.1038/s41419-019-1716-9 PMC6586814

[127] Huang H (2020). Pericyte-Endothelial Interactions in the Retinal Microvasculature. Int J Mol Sci, 21. 10.3390/ijms21197413 PMC7582747 33049983

[128] Sweeney MD, Ayyadurai S, Zlokovic BV (2016). Pericytes of the neurovascular unit: key functions and signaling pathways. Nat Neurosci, 19:771-783. 27227366 10.1038/nn.4288 PMC5745011

[129] Fisher M (2009). Pericyte signaling in the neurovascular unit. Stroke, 40:S13-15. 19064799 10.1161/STROKEAHA.108.533117 PMC2724312

[130] Cai W, Zhang K, Li P, Zhu L, Xu J, Yang B, et al. (2017). Dysfunction of the neurovascular unit in ischemic stroke and neurodegenerative diseases: An aging effect. Ageing Res Rev, 34:77-87. 27697546 10.1016/j.arr.2016.09.006 PMC5384332

[131] Yang S, Jin H, Zhu Y, Wan Y, Opoku EN, Zhu L, et al. (2017). Diverse Functions and Mechanisms of Pericytes in Ischemic Stroke. Curr Neuropharmacol, 15:892-905. 28088914 10.2174/1570159X15666170112170226 PMC5652032

[132] Quelhas P, Baltazar G, Cairrao E (2019). The Neurovascular Unit: Focus on the Regulation of Arterial Smooth Muscle Cells. Curr Neurovasc Res, 16:502-515. 31738142 10.2174/1567202616666191026122642

[133] Durham AL, Speer MY, Scatena M, Giachelli CM, Shanahan CM (2018). Role of smooth muscle cells in vascular calcification: implications in atherosclerosis and arterial stiffness. Cardiovasc Res, 114:590-600. 29514202 10.1093/cvr/cvy010 PMC5852633

[134] Poittevin M, Lozeron P, Hilal R, Levy BI, Merkulova-Rainon T, Kubis N (2014). Smooth muscle cell phenotypic switching in stroke. Transl Stroke Res, 5:377-384. 24323725 10.1007/s12975-013-0306-x

[135] Mastorakos P, McGavern D (2019). The anatomy and immunology of vasculature in the central nervous system. Sci Immunol, 4. 10.1126/sciimmunol.aav0492 PMC6816468 31300479

[136] Vanlandewijck M, He L, Mae MA, Andrae J, Ando K, Del Gaudio F, et al. (2018). A molecular atlas of cell types and zonation in the brain vasculature. Nature, 554:475-480. 29443965 10.1038/nature25739

[137] Thulabandu V, Chen D, Atit RP (2018). Dermal fibroblast in cutaneous development and healing. Wiley Interdiscip Rev Dev Biol, 7. 10.1002/wdev.307 PMC5814349 29244903

[138] Gurtner GC, Werner S, Barrandon Y, Longaker MT (2008). Wound repair and regeneration. Nature, 453:314-321. 18480812 10.1038/nature07039

[139] Riew TR, Choi JH, Kim HL, Jin X, Lee MY (2018). PDGFR-beta-Positive Perivascular Adventitial Cells Expressing Nestin Contribute to Fibrotic Scar Formation in the Striatum of 3-NP Intoxicated Rats. Front Mol Neurosci, 11:402. 30455628 10.3389/fnmol.2018.00402 PMC6230557

[140] Fernandez-Klett F, Potas JR, Hilpert D, Blazej K, Radke J, Huck J, et al. (2013). Early loss of pericytes and perivascular stromal cell-induced scar formation after stroke. J Cereb Blood Flow Metab, 33:428-439. 23250106 10.1038/jcbfm.2012.187 PMC3587816

[141] Kelly KK, MacPherson AM, Grewal H, Strnad F, Jones JW, Yu J, et al. (2016). Col1a1+ perivascular cells in the brain are a source of retinoic acid following stroke. BMC Neurosci, 17:49. 27422020 10.1186/s12868-016-0284-5 PMC4947279

[142] Ma Q, Huang B, Khatibi N, Rolland W 2nd, Suzuki H, Zhang JH, et al. (2011). PDGFR-alpha inhibition preserves blood-brain barrier after intracerebral hemorrhage. Ann Neurol, 70:920-931. 22190365 10.1002/ana.22549 PMC3405848

[143] Su EJ, Fredriksson L, Geyer M, Folestad E, Cale J, Andrae J, et al. (2008). Activation of PDGF-CC by tissue plasminogen activator impairs blood-brain barrier integrity during ischemic stroke. Nat Med, 14:731-737. 18568034 10.1038/nm1787 PMC2811427

[144] Zera KA, Buckwalter MS (2020). The Local and Peripheral Immune Responses to Stroke: Implications for Therapeutic Development. Neurotherapeutics, 17:414-435. 32193840 10.1007/s13311-020-00844-3 PMC7283378

[145] Kang L, Yu H, Yang X, Zhu Y, Bai X, Wang R, et al. (2020). Neutrophil extracellular traps released by neutrophils impair revascularization and vascular remodeling after stroke. Nat Commun, 11:2488. 32427863 10.1038/s41467-020-16191-y PMC7237502

[146] Enzmann G, Kargaran S, Engelhardt B (2018). Ischemia-reperfusion injury in stroke: impact of the brain barriers and brain immune privilege on neutrophil function. Ther Adv Neurol Disord, 11:1756286418794184. 30181779 10.1177/1756286418794184 PMC6111395

[147] Jickling GC, Liu D, Ander BP, Stamova B, Zhan X, Sharp FR (2015). Targeting neutrophils in ischemic stroke: translational insights from experimental studies. J Cereb Blood Flow Metab, 35:888-901. 25806703 10.1038/jcbfm.2015.45 PMC4640255

[148] Winneberger J, Schols S, Lessmann K, Randez-Garbayo J, Bauer AT, Mohamud Yusuf A, et al. (2021). Platelet endothelial cell adhesion molecule-1 is a gatekeeper of neutrophil transendothelial migration in ischemic stroke. Brain Behav Immun, 93:277-287. 33388423 10.1016/j.bbi.2020.12.026

[149] Gill D, Sivakumaran P, Aravind A, Tank A, Dosh R, Veltkamp R (2018). Temporal Trends in the Levels of Peripherally Circulating Leukocyte Subtypes in the Hours after Ischemic Stroke. J Stroke Cerebrovasc Dis, 27:198-202. 28927686 10.1016/j.jstrokecerebrovasdis.2017.08.023

[150] Tang C, Wang C, Zhang Y, Xue L, Li Y, Ju C, et al. (2019). Recognition, Intervention, and Monitoring of Neutrophils in Acute Ischemic Stroke. Nano Lett, 19:4470-4477. 31244234 10.1021/acs.nanolett.9b01282

[151] Perez-de-Puig I, Miro-Mur F, Ferrer-Ferrer M, Gelpi E, Pedragosa J, Justicia C, et al. (2015). Neutrophil recruitment to the brain in mouse and human ischemic stroke. Acta Neuropathol, 129:239-257. 25548073 10.1007/s00401-014-1381-0

[152] Guldolf K, Vandervorst F, Gens R, Ourtani A, Scheinok T, De Raedt S (2021). Neutrophil-to-lymphocyte ratio predicts delirium after stroke. Age Ageing, 50:1626-1632. 34218276 10.1093/ageing/afab133

[153] Laridan E, Denorme F, Desender L, Francois O, Andersson T, Deckmyn H, et al. (2017). Neutrophil extracellular traps in ischemic stroke thrombi. Ann Neurol, 82:223-232. 28696508 10.1002/ana.24993

[154] Cuartero MI, Ballesteros I, Moraga A, Nombela F, Vivancos J, Hamilton JA, et al. (2013). N2 neutrophils, novel players in brain inflammation after stroke: modulation by the PPARgamma agonist rosiglitazone. Stroke, 44:3498-3508. 24135932 10.1161/STROKEAHA.113.002470

[155] Cai W, Liu S, Hu M, Huang F, Zhu Q, Qiu W, et al. (2020). Functional Dynamics of Neutrophils After Ischemic Stroke. Transl Stroke Res, 11:108-121. 30847778 10.1007/s12975-019-00694-y PMC6993940

[156] Garcia-Culebras A, Duran-Laforet V, Pena-Martinez C, Moraga A, Ballesteros I, Cuartero MI, et al. (2019). Role of TLR4 (Toll-Like Receptor 4) in N1/N2 Neutrophil Programming After Stroke. Stroke, 50:2922-2932. 31451099 10.1161/STROKEAHA.119.025085

[157] Easton AS (2013). Neutrophils and stroke - can neutrophils mitigate disease in the central nervous system? Int Immunopharmacol, 17:1218-1225. 23827753 10.1016/j.intimp.2013.06.015

[158] Hou Y, Yang D, Xiang R, Wang H, Wang X, Zhang H, et al. (2019). N2 neutrophils may participate in spontaneous recovery after transient cerebral ischemia by inhibiting ischemic neuron injury in rats. Int Immunopharmacol, 77:105970. 31675618 10.1016/j.intimp.2019.105970

[159] Xie X, Shi Q, Wu P, Zhang X, Kambara H, Su J, et al. (2020). Single-cell transcriptome profiling reveals neutrophil heterogeneity in homeostasis and infection. Nat Immunol, 21:1119-1133. 32719519 10.1038/s41590-020-0736-z PMC7442692

[160] Wanrooy BJ, Wen SW, Wong CH (2021). Dynamic roles of neutrophils in post-stroke neuroinflammation. Immunol Cell Biol, 99:924-935. 33894069 10.1111/imcb.12463

[161] Zhu YP, Padgett L, Dinh HQ, Marcovecchio P, Blatchley A, Wu R, et al. (2018). Identification of an Early Unipotent Neutrophil Progenitor with Pro-tumoral Activity in Mouse and Human Bone Marrow. Cell Rep, 24:2329-2341 e2328. 30157427 10.1016/j.celrep.2018.07.097 PMC6542273

[162] Watzlawick R, Kenngott EE, Liu FD, Schwab JM, Hamann A (2015). Anti-Inflammatory Effects of IL-27 in Zymosan-Induced Peritonitis: Inhibition of Neutrophil Recruitment Partially Explained by Impaired Mobilization from Bone Marrow and Reduced Chemokine Levels. PLoS One, 10:e0137651. 26360023 10.1371/journal.pone.0137651 PMC4567321

[163] Meng H, Zhao H, Cao X, Hao J, Zhang H, Liu Y, et al. (2019). Double-negative T cells remarkably promote neuroinflammation after ischemic stroke. Proc Natl Acad Sci U S A, 116:5558-5563. 30819895 10.1073/pnas.1814394116 PMC6431175

[164] Iadecola C, Anrather J (2011). The immunology of stroke: from mechanisms to translation. Nat Med, 17:796-808. 21738161 10.1038/nm.2399 PMC3137275

[165] Shichita T, Sugiyama Y, Ooboshi H, Sugimori H, Nakagawa R, Takada I, et al. (2009). Pivotal role of cerebral interleukin-17-producing gammadeltaT cells in the delayed phase of ischemic brain injury. Nat Med, 15:946-950. 19648929 10.1038/nm.1999

[166] Loetscher P, Pellegrino A, Gong JH, Mattioli I, Loetscher M, Bardi G, et al. (2001). The ligands of CXC chemokine receptor 3, I-TAC, Mig, and IP10, are natural antagonists for CCR3. J Biol Chem, 276:2986-2991. 11110785 10.1074/jbc.M005652200

[167] Angiari S, Donnarumma T, Rossi B, Dusi S, Pietronigro E, Zenaro E, et al. (2014). TIM-1 glycoprotein binds the adhesion receptor P-selectin and mediates T cell trafficking during inflammation and autoimmunity. Immunity, 40:542-553. 24703780 10.1016/j.immuni.2014.03.004 PMC4066214

[168] Ito M, Komai K, Mise-Omata S, Iizuka-Koga M, Noguchi Y, Kondo T, et al. (2019). Brain regulatory T cells suppress astrogliosis and potentiate neurological recovery. Nature, 565:246-250. 30602786 10.1038/s41586-018-0824-5

[169] Miro-Mur F, Urra X, Ruiz-Jaen F, Pedragosa J, Chamorro A, Planas AM (2020). Antigen-Dependent T Cell Response to Neural Peptides After Human Ischemic Stroke. Front Cell Neurosci, 14:206. 32719588 10.3389/fncel.2020.00206 PMC7348665

[170] Wang H, Wang Z, Wu Q, Yuan Y, Cao W, Zhang X (2021). Regulatory T cells in ischemic stroke. CNS Neurosci Ther, 27:643-651. 33470530 10.1111/cns.13611 PMC8111493

[171] Hu Y, Zheng Y, Wu Y, Ni B, Shi S (2014). Imbalance between IL-17A-producing cells and regulatory T cells during ischemic stroke. Mediators Inflamm, 2014:813045. 24991091 10.1155/2014/813045 PMC4058812

[172] Li P, Mao L, Liu X, Gan Y, Zheng J, Thomson AW, et al. (2014). Essential role of program death 1-ligand 1 in regulatory T-cell-afforded protection against blood-brain barrier damage after stroke. Stroke, 45:857-864. 24496394 10.1161/STROKEAHA.113.004100 PMC3939692

[173] Stubbe T, Ebner F, Richter D, Engel O, Klehmet J, Royl G, et al. (2013). Regulatory T cells accumulate and proliferate in the ischemic hemisphere for up to 30 days after MCAO. J Cereb Blood Flow Metab, 33:37-47. 22968321 10.1038/jcbfm.2012.128 PMC3597367

[174] Kleinschnitz C, Kraft P, Dreykluft A, Hagedorn I, Gobel K, Schuhmann MK, et al. (2013). Regulatory T cells are strong promoters of acute ischemic stroke in mice by inducing dysfunction of the cerebral microvasculature. Blood, 121:679-691. 23160472 10.1182/blood-2012-04-426734 PMC3790947

[175] Pruss H, Iggena D, Baldinger T, Prinz V, Meisel A, Endres M, et al. (2012). Evidence of intrathecal immunoglobulin synthesis in stroke: a cohort study. Arch Neurol, 69:714-717. 22371852 10.1001/archneurol.2011.3252

[176] Chen Y, Bodhankar S, Murphy SJ, Vandenbark AA, Alkayed NJ, Offner H (2012). Intrastriatal B-cell administration limits infarct size after stroke in B-cell deficient mice. Metab Brain Dis, 27:487-493. 22618587 10.1007/s11011-012-9317-7 PMC3427715

[177] Ren X, Akiyoshi K, Dziennis S, Vandenbark AA, Herson PS, Hurn PD, et al. (2011). Regulatory B cells limit CNS inflammation and neurologic deficits in murine experimental stroke. J Neurosci, 31:8556-8563. 21653859 10.1523/JNEUROSCI.1623-11.2011 PMC3111929

[178] Kitamura D, Roes J, Kuhn R, Rajewsky K (1991). A B cell-deficient mouse by targeted disruption of the membrane exon of the immunoglobulin mu chain gene. Nature, 350:423-426. 1901381 10.1038/350423a0

[179] Ortega SB, Torres VO, Latchney SE, Whoolery CW, Noorbhai IZ, Poinsatte K, et al. (2020). B cells migrate into remote brain areas and support neurogenesis and functional recovery after focal stroke in mice. Proc Natl Acad Sci U S A, 117:4983-4993. 32051245 10.1073/pnas.1913292117 PMC7060723

[180] Levine DA, Galecki AT, Langa KM, Unverzagt FW, Kabeto MU, Giordani B, et al. (2015). Trajectory of Cognitive Decline After Incident Stroke. JAMA, 314:41-51. 26151265 10.1001/jama.2015.6968 PMC4655087

[181] Doyle KP, Quach LN, Sole M, Axtell RC, Nguyen TV, Soler-Llavina GJ, et al. (2015). B-lymphocyte-mediated delayed cognitive impairment following stroke. J Neurosci, 35:2133-2145. 25653369 10.1523/JNEUROSCI.4098-14.2015 PMC4315838

[182] Becker KJ, Tanzi P, Zierath D, Buckwalter MS (2016). Antibodies to myelin basic protein are associated with cognitive decline after stroke. J Neuroimmunol, 295-296:9-11. 27235342 10.1016/j.jneuroim.2016.04.001 PMC4884610

[183] Pendlebury ST, Rothwell PM (2009). Prevalence, incidence, and factors associated with pre-stroke and post-stroke dementia: a systematic review and meta-analysis. Lancet Neurol, 8:1006-1018. 19782001 10.1016/S1474-4422(09)70236-4

[184] Beli E, Clinthorne JF, Duriancik DM, Hwang I, Kim S, Gardner EM (2011). Natural killer cell function is altered during the primary response of aged mice to influenza infection. Mech Ageing Dev, 132:503-510. 21893080 10.1016/j.mad.2011.08.005 PMC3185116

[185] Crinier A, Narni-Mancinelli E, Ugolini S, Vivier E (2020). SnapShot: Natural Killer Cells. Cell, 180:1280-1280 e1281. 32200803 10.1016/j.cell.2020.02.029

[186] Gan Y, Liu Q, Wu W, Yin JX, Bai XF, Shen R, et al. (2014). Ischemic neurons recruit natural killer cells that accelerate brain infarction. Proc Natl Acad Sci U S A, 111:2704-2709. 24550298 10.1073/pnas.1315943111 PMC3932858

[187] Liu Q, Jin WN, Liu Y, Shi K, Sun H, Zhang F, et al. (2017). Brain Ischemia Suppresses Immunity in the Periphery and Brain via Different Neurogenic Innervations. Immunity, 46:474-487. 28314594 10.1016/j.immuni.2017.02.015

[188] Souza-Fonseca-Guimaraes F, Parlato M, Philippart F, Misset B, Cavaillon JM, Adib-Conquy M, et al. (2012). Toll-like receptors expression and interferon-gamma production by NK cells in human sepsis. Crit Care, 16:R206. 23098236 10.1186/cc11838 PMC3682310

[189] Duran-Laforet V, Pena-Martinez C, Garcia-Culebras A, Alzamora L, Moro MA, Lizasoain I (2021). Pathophysiological and pharmacological relevance of TLR4 in peripheral immune cells after stroke. Pharmacol Ther, 228:107933. 34174279 10.1016/j.pharmthera.2021.107933

[190] Tushev G, Glock C, Heumuller M, Biever A, Jovanovic M, Schuman EM (2018). Alternative 3' UTRs Modify the Localization, Regulatory Potential, Stability, and Plasticity of mRNAs in Neuronal Compartments. Neuron, 98:495-511 e496. 29656876 10.1016/j.neuron.2018.03.030

[191] Cajigas IJ, Tushev G, Will TJ, tom Dieck S, Fuerst N, Schuman EM (2012). The local transcriptome in the synaptic neuropil revealed by deep sequencing and high-resolution imaging. Neuron, 74:453-466. 22578497 10.1016/j.neuron.2012.02.036 PMC3627340

[192] Bakken TE, Hodge RD, Miller JA, Yao Z, Nguyen TN, Aevermann B, et al. (2018). Single-nucleus and single-cell transcriptomes compared in matched cortical cell types. PLoS One, 13:e0209648. 30586455 10.1371/journal.pone.0209648 PMC6306246

[193] Tasic B, Yao Z, Graybuck LT, Smith KA, Nguyen TN, Bertagnolli D, et al. (2018). Shared and distinct transcriptomic cell types across neocortical areas. Nature, 563:72-78. 30382198 10.1038/s41586-018-0654-5 PMC6456269

[194] Lacar B, Linker SB, Jaeger BN, Krishnaswami SR, Barron JJ, Kelder MJE, et al. (2016). Nuclear RNA-seq of single neurons reveals molecular signatures of activation. Nat Commun, 7:11022. 27090946 10.1038/ncomms11022 PMC4838832

[195] Bergen V, Lange M, Peidli S, Wolf FA, Theis FJ (2020). Generalizing RNA velocity to transient cell states through dynamical modeling. Nat Biotechnol, 38:1408-1414. 32747759 10.1038/s41587-020-0591-3

[196] Savulescu AF, Jacobs C, Negishi Y, Davignon L, Mhlanga MM (2020). Pinpointing Cell Identity in Time and Space. Front Mol Biosci, 7:209. 32923457 10.3389/fmolb.2020.00209 PMC7456825

[197] Welch JD, Kozareva V, Ferreira A, Vanderburg C, Martin C, Macosko EZ (2019). Single-Cell Multi-omic Integration Compares and Contrasts Features of Brain Cell Identity. Cell, 177:1873-1887 e1817. 31178122 10.1016/j.cell.2019.05.006 PMC6716797

[198] Pedroza AJ, Tashima Y, Shad R, Cheng P, Wirka R, Churovich S, et al. (2020). Single-Cell Transcriptomic Profiling of Vascular Smooth Muscle Cell Phenotype Modulation in Marfan Syndrome Aortic Aneurysm. Arterioscler Thromb Vasc Biol, 40:2195-2211. 32698686 10.1161/ATVBAHA.120.314670 PMC7484233

[199] Maynard KR, Collado-Torres L, Weber LM, Uytingco C, Barry BK, Williams SR, et al. (2021). Transcriptome-scale spatial gene expression in the human dorsolateral prefrontal cortex. Nat Neurosci, 24:425-436. 33558695 10.1038/s41593-020-00787-0 PMC8095368

